# Nature meets machine: the AI renaissance in natural product drug discovery

**DOI:** 10.1007/s13659-025-00589-6

**Published:** 2026-03-02

**Authors:** Rajesh Muthuraj, Jaikanth Chandrasekaran

**Affiliations:** Department of Pharmacology, Sri Ramachandra Faculty of Pharmacy, Sri Ramachandra Institute of Higher Education and Research (Deemed to Be University), Chennai, 600116 Tamil Nadu India

**Keywords:** Natural products (NPs), Artificial intelligence (AI), Machine learning, Drug discovery, Cheminformatics, Dereplication, Traditional medicine, Ethnopharmacology

## Abstract

Natural products (NPs) have long served as a cornerstone of drug discovery, yielding landmark therapeutics such as paclitaxel and artemisinin and providing sustained access to biologically relevant chemical space. Despite this legacy, NP-based discovery has gradually declined with the rise of synthetic chemistry and high-throughput screening, even as many contemporary “synthetic” drugs remain structurally inspired by natural scaffolds. Classical NP workflows—centered on phenotypic screening and bioassay-guided fractionation—continue to face persistent bottlenecks, including structural complexity, low bioactive yield, frequent rediscovery, and limited scalability. Rather than competing with NP research, artificial intelligence (AI) offers a complementary methodological framework to address these longstanding challenges. This review critically examines the bottlenecks inherent to traditional NP discovery and outlines how AI can be systematically integrated across the pipeline. We discuss AI-enabled advances ranging from natural language processing for mining ethnopharmacological knowledge to machine learning–driven dereplication, cheminformatics, and genome mining, with platforms such as GNPS2 exemplifying scalable progress. Case studies in antibiotic and anticancer discovery, as well as the modernization of traditional medicine, illustrate how AI–NP integration can accelerate early-stage discovery while enhancing translational relevance. Looking ahead, we examine emerging paradigms—including quantum machine learning, federated data ecosystems, and AI-assisted molecular design—that may further expand the scope of NP-based research. Collectively, this review presents a forward-looking framework in which AI functions not as a replacement for NP science, but as a synergistic discipline that enables more efficient, scalable, and informed exploration of nature-derived chemical diversity.

**Graphical Abstract:**

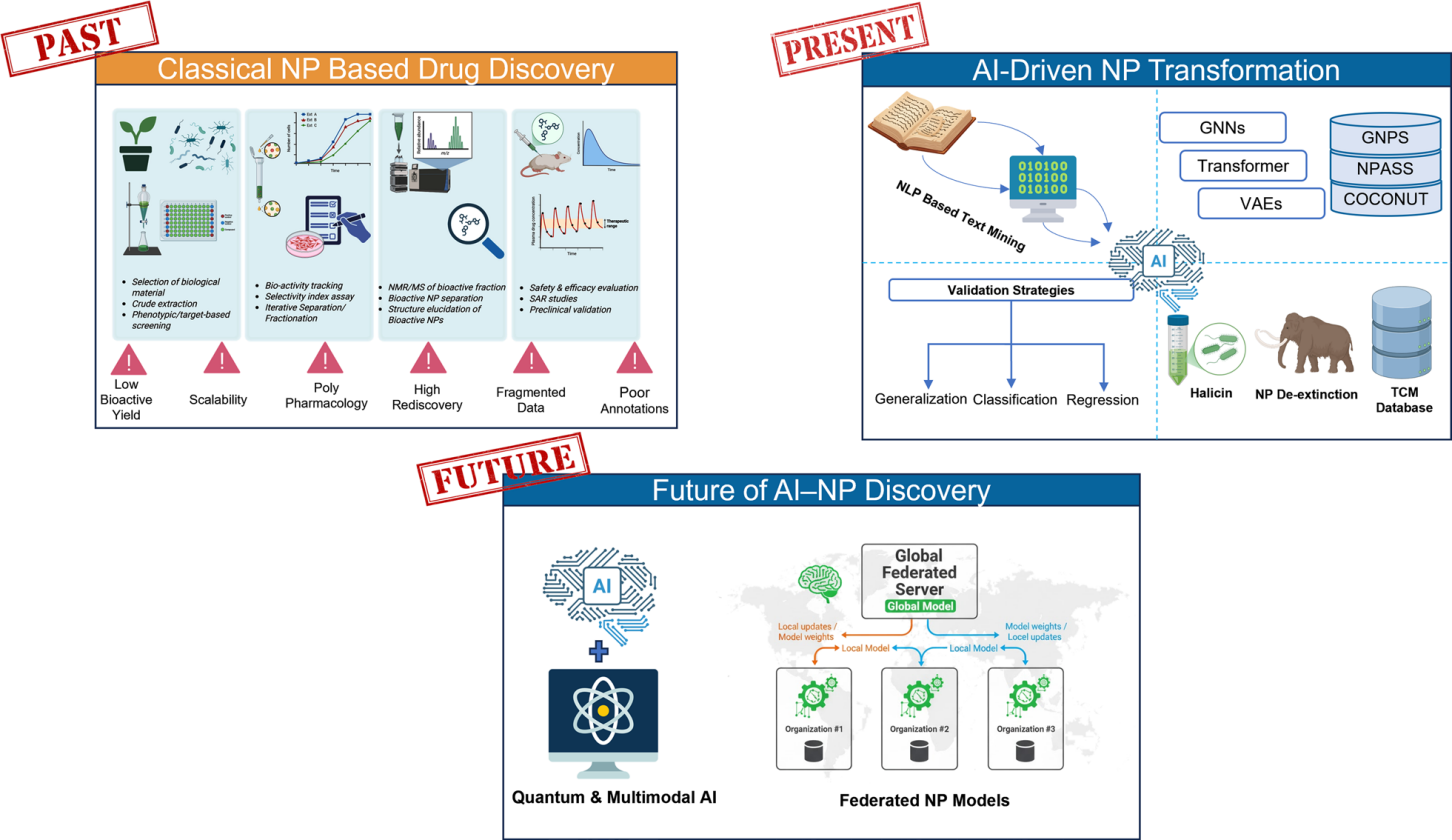

**Supplementary Information:**

The online version contains supplementary material available at 10.1007/s13659-025-00589-6.

## Introduction

The exploration of nature’s chemical diversity has underpinned human survival, the evolution of medicine, and the advancement of Pharmacological science. Medicinal plants, in particular, have served as an enduring source of therapeutic agents across civilizations, shaping both traditional systems of healing and the foundations of modern drug discovery. From early herbal remedies to contemporary small-molecule therapeutics, plant-derived Natural Products (NP) have played a pivotal role—especially in the treatment of complex diseases such as cancer.

Traditional medicine systems across civilizations—including Ayurveda, Traditional Chinese Medicine, and Indigenous healing practices—have documented the therapeutic use of natural products for millennia [1–4]. These early pharmacopeias laid the groundwork for global therapeutic frameworks. Despite the synthetic drug revolution of the twentieth century, plant-based medicines have retained relevance. The COVID-19 pandemic, for example, renewed global interest in botanical therapeutics, prompting a surge of research into bioactive compounds embedded in traditional practices [5, 6]. However, modern biomedicine often views traditional remedies with skepticism—a reflection not of their historical ineffectiveness but of the methodological challenges in validating them. Traditional formulations, often chemically complex, demand phenotypic-based screening to identify active principles, followed by rigorous randomized controlled trials (RCTs) to ensure reproducibility and safety before integration into modern pharmacopoeias.

Among natural products, plant-derived secondary metabolites remain a particularly rich source of Pharmacological innovation. Unlike primary metabolites essential for cellular function, secondary metabolites—such as alkaloids, flavonoids, terpenoids, Glycosides, Saponins and polyphenols—serve ecological functions like defence and stress adaptation, and have demonstrated potent bioactivity across therapeutic classes [7, 8]. Among these, alkaloids (e.g., morphine, vincristine) have yielded landmark analgesics and anticancer agents, while terpenoids (e.g., artemisinin, paclitaxel) have provided antimalarial and cytotoxic scaffolds of profound clinical importance [9, 10]. Their structural diversity and biological specificity have consistently inspired drug development, particularly in oncology. A landmark review of drug approvals over four decades revealed that between 1946 and 1980, over 50% of cancer drugs were natural products or their derivatives, and from 1981 onwards, natural scaffolds accounted for nearly two-thirds of small-molecule anticancer drugs when semi-synthetics are included [11]. Yet, despite this impressive legacy, enthusiasm for NP-based drug discovery has waned in recent decades [12]. A range of technical and logistical barriers has contributed to this decline: interference of complex extracts in high-throughput screening (HTS) assays, frequent rediscovery of known molecules, low natural abundance of active constituents, and the presence of synergistic or nuisance compounds that complicate mechanistic deconvolution. Further, evolving legal frameworks such as the Convention on Biological Diversity (CBD) and the Nagoya Protocol have introduced regulatory friction in global bioprospecting.

The convergence of Artificial Intelligence (AI) and natural product science represents a paradigm shift. To fully realize its potential, it will require sustained collaboration between natural product chemists, Pharmacologists, AI engineers, and drug discovery scientists. This union could not only accelerate the discovery of novel plant-derived therapeutics but also create an evidence-based bridge between traditional medicine and modern pharmacology.

In this Review, we examine the structural, pharmacological, and logistical limitations of classical NP discovery pipelines and outline a forward-looking, AI-enabled framework for plant-based drug discovery. We highlight emerging AI and machine learning tools with relevance to NP research, discuss key ethical and regulatory considerations, and introduce next-generation computational technologies—including quantum machine learning—that could further substantially advance the search for plant-derived bioactive leads.

## The decline of natural products in pharmaceutical innovation: causes and consequences

NP, particularly those derived from plants have long served as critical starting points and lead compounds in drug discovery [11, 13]. Historically overlooked as mere metabolic byproducts, plant secondary metabolites were largely dismissed until the early nineteenth century, when Friedrich Wilhelm Sertürner achieved the first isolation of morphine from the opium poppy (*Papaver somniferum*) [14, 15]. This landmark discovery demonstrated, for the first time, that the therapeutic effects of a medicinal plant could be attributed to a single, chemically defined compound. Sertürner’s work not only marked the formal birth of NP-based pharmaceutical research but also catalyzed the rise of pharmacognosy and the systematic study of natural compound chemistry [15].

**Fig. 1:**
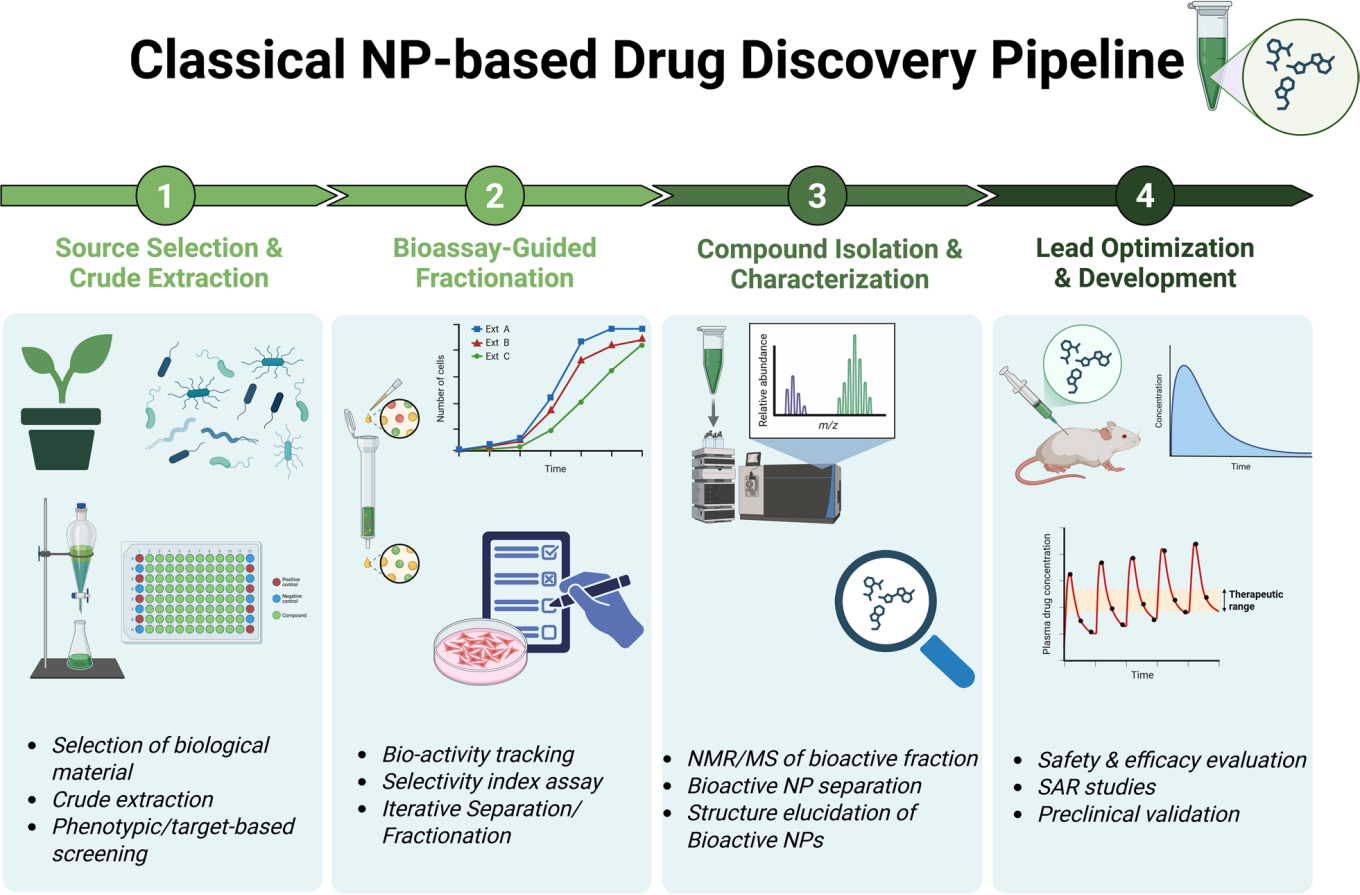
Classical natural product–based drug discovery pipeline. A traditional NP-based drug discovery workflow typically begins with the selection of a biomaterial, often guided by phenotypic screening or ethnopharmacological evidence. This is followed by rigorous bioassay-guided fractionation, isolation of active principles, and structural characterization using analytical techniques. The process culminates in lead development and preclinical validation of therapeutic efficacy and safety

The classical NP discovery pipeline has traditionally relied on phenotypic screening approaches, in which compounds are evaluated in biological systems based on their ability to elicit desirable cellular or organismal responses *(*Fig. 1*)*. This target-agnostic strategy, unbiased by prior mechanistic assumptions has proven especially fruitful in identifying “first-in-class” therapeutics with novel modes of action [16].

Plant-derived NPs possess a unique constellation of properties: high biological activity, structural diversity, chemical complexity, and favorable biocompatibility. These features often surpass those found in fully synthetic molecules and enable NPs to occupy underexplored regions of chemical space, offering creative scaffolds for medicinal chemistry innovation [17]. Such attributes have been particularly advantageous in the development of therapies for cancer, infectious diseases, and chronic inflammatory conditions, where synthetic libraries have often fallen short [18–20].

**Fig. 2:**
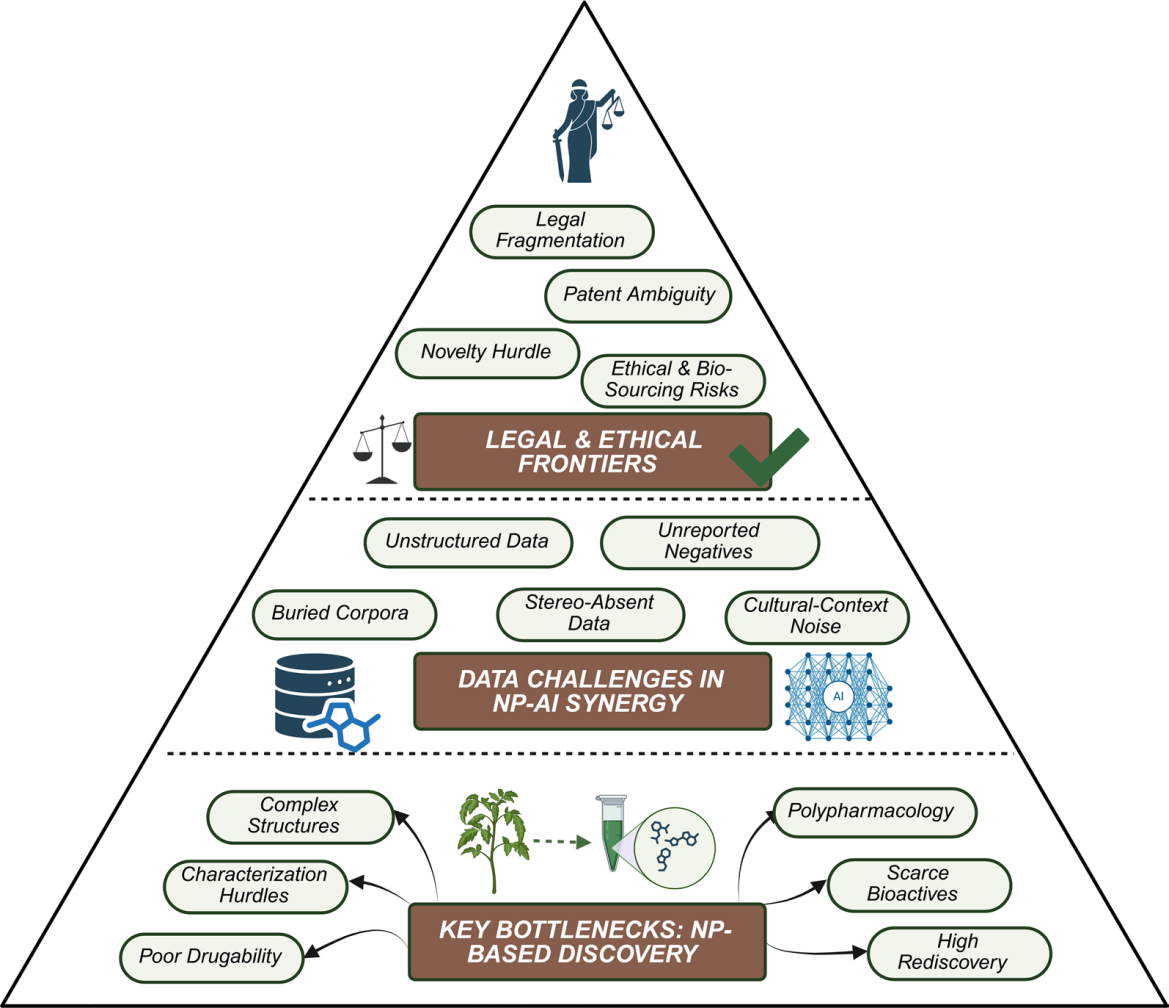
Major challenges in natural product–based drug discovery. This figure summarizes key obstacles across the NP drug discovery pipeline, including issues related to compound dereplication, structural complexity, polypharmacology, intellectual property constraints, and the integration of heterogeneous datasets

However, despite their historical significance and continued pharmacological promise, NP-based drug discovery has experienced a notable decline in recent decades [20, 21]. These challenges are multifactorial, ranging from difficulties in compound isolation and characterization to issues with reproducibility, supply chain limitations, and the rediscovery of known molecules. In the sections that follow, we examine the major factors underlying this downturn. First, we assess the core limitations of the traditional NP discovery pipeline, including technical, logistical, and chemical challenges. We then explore the data-related hurdles that have impeded the effective application of AI and Machine learning (ML) to NP research. Finally, we discuss the evolving legal and ethical landscape surrounding natural product sourcing (Fig. 2).

### NP-specific challenges

The drug discovery potential of NPs, particularly those derived from plants, is fundamentally shaped and often limited by their unique chemical and biological characteristics. While these compounds offer high structural diversity and bioactivity, their inherent complexity introduces several critical challenges across the NP discovery pipeline. Chief among these are difficulties related to structural elucidation, low natural abundance, inefficient extraction and modification of workflows, persistent issues with dereplication, and unfavorable physicochemical properties (Table 1). Collectively, these factors hinder the efficient translation of bioactive NPs into clinically viable therapeutics and contribute to the broader decline in NP-based pharmaceutical innovation.

**Table 1:** Key challenges in NP drug discovery and AI-enabled solutions

NP challenges	Description	Impact on NP based drug discovery	Potential solutions	AI-enabled approaches
Complex Structural Elucidation	NPs often exhibit highly complex, stereo chemically rich structures that challenge conventional NMR and MS workflows	Labor-intensive and time-consuming elucidation slows down lead identification and validation	Machine learning-assisted NMR/MS interpretation; CASE systems; AI-driven structure prediction tools	ML-based spectral deconvolution; probabilistic structure ranking
Low Abundance of Bioactives	Bioactive compounds are frequently present in trace amounts and may be lost during classical extraction or fractionation	Missed actives may result in false negatives during bioassays, reducing hit rates	AI/ML-based extract ranking and guided fractionation; integration of omics-guided prioritization methods	Predictive extract prioritization; AI-guided bioactivity prediction
Poor Druggability	Many NPs have suboptimal ADMET properties, including poor solubility, bioavailability, or metabolic stability	Hinders development into viable drug candidates and leads to lower industry adoption of NP scaffolds	AI-assisted scaffold optimization; early-stage filtering for drug-likeness; property-guided molecular editing	Generative models with built-in ADMET filtering
Polypharmacology	A single NP may modulate multiple targets, leading to both desired multitarget effects and undesirable off-target actions	Increases complexity in target deconvolution and MOA elucidation	AI-powered target prediction; network pharmacology; multi-omics integration	Integration of proteomic tools (e.g., TPP, PISA); AI-based target deconvolution frameworks
High Rate of Rediscovery	Classical workflows often re-identify known compounds, reducing novelty and consuming resources	Diminishes researcher motivation and delays access to novel scaffolds for development	AI-enhanced dereplication pipelines (e.g., GNPS, SMART 2.0); spectral library integration	Automated dereplication using AI-driven spectral matching and similarity scoring

#### Structural complexity and challenges in elucidation

NP discovery typically begins with the extraction and isolation of bioactive constituents, most commonly plant-derived secondary metabolites through bioassay-guided fractionation. This is followed by structure elucidation using advanced analytical tools such as Nuclear magnetic resonance (NMR) spectroscopy, mass spectrometry, and X-ray crystallography [22]. These steps, though essential, are labor-intensive, time-consuming, and resource-intensive. A well-known example is the anticancer agent paclitaxel (Taxol), isolated from the Pacific yew tree (*Taxus brevifolia*), which required more than three decades of research to move from initial discovery to clinical application [23].

The principal bottleneck lies in the intrinsic structural complexity of many NPs. These compounds often contain multiple stereogenic centers, highly functionalized ring systems, sp^3^-rich carbon frameworks, numerous hydrogen bond donors and acceptors, and conformational flexibility due to rotatable bonds [17]. While such features enhance biological activity and increase the likelihood of engaging challenging targets, they also complicate synthetic optimization and medicinal chemistry campaigns.

Moreover, the polypharmacological nature of many NPs—where a single compound interacts with multiple biological targets—can be both an asset and a liability. On one hand, this multi-target activity may be beneficial for treating complex diseases such as cancer or neurodegeneration. On the other, it raises the risk of off-target effects and toxicity, complicates mechanism-of-action (MOA) studies, and makes target deconvolution significantly more difficult than in single-target synthetic drug frameworks [24–27].

#### Scarcity and challenges in compound isolation

Another longstanding hurdle is the typically low natural abundance of many bioactive NPs. These compounds often occur in trace quantities—ranging from micrograms to a few milligrams per gram of plant material—rendering conventional extraction inefficient, environmentally unsustainable, and economically burdensome [28]. Such scarcity imposes major constraints on early-stage phenotypic screening and downstream pharmacological validation. Additionally, attempts to synthetically modify these complex scaffolds often result in low-yield reactions, mixtures of poorly separable analogs, or difficult-to-purify intermediates, further limiting the ability to generate high-purity derivatives in quantities sufficient for lead optimization [29].

#### Dereplication and the rediscovery problem

Rediscovery of already well characterised compounds is big issue in the NP based drug discovery. Dereplication, the early identification and exclusion of previously characterized compounds is a very complex process in NP drug discovery. Traditional chromatographic and spectrometric workflows frequently lead to the rediscovery of known metabolites, draining resources and reducing the novelty yield of screening campaigns [21, 30]. While dereplication technologies have improved in recent years, especially with the incorporation of spectral libraries and informatics tools, HTS platforms remain poorly suited to NP mixtures. The complex and variable composition of NP extracts can interfere with assay fidelity, complicate automation, and obscure the identification of novel active constituents—even in AI-assisted or robotics-enhanced workflows.

#### Physicochemical and pharmacokinetic limitations

In addition to structural and workflow-related barriers, many NPs exhibit suboptimal physicochemical and pharmacokinetic properties. Common issues include poor aqueous solubility, limited metabolic stability, low oral bioavailability, and dose-limiting toxicity [12, 31]. These properties undermine formulation development and impede clinical translation. Although medicinal chemistry can sometimes address these shortcomings, the structural complexity of NPs makes such optimization especially challenging and resource-intensive.

### Data Challenges in NP–AI Synergy

The successful integration of AI and ML into NP-based drug discovery hinges critically on the availability of large, high-quality, and well-annotated datasets. However, the current NP research ecosystem suffers from a range of data-related limitations that constrain model performance, hinder reproducibility, and undermine generalizability (Table 2). A fundamental challenge is the fragmentation and lack of standardization across NP-related databases. Many existing repositories suffer from infrequent updates, inconsistent data formats, and limited support for advanced querying tools such as substructure and similarity searches [32, 33]. Crucial stereochemical information— essential for modeling molecular interactions with biological targets—is often missing, ambiguously encoded, or inconsistently represented, thereby compromising the accuracy of downstream computational analyses. Publicly available NP databases are frequently incomplete, unevenly annotated, or contain erroneous entries, making them suboptimal as training sets for ML models [34]. In addition, these resources often lack rich metadata necessary for contextual learning, including information on experimental design, assay conditions, organismal sources, extraction methods, and fraction-level bioactivity data. The absence of such metadata hampers the development of context-aware models capable of accurately predicting biological responses or target interactions. Another critical limitation is the scarcity of published negative results. The absence of such data introduces systematic bias in training sets, predisposing models to overfitting and inflating performance metrics during benchmarking [35]. This lack of balanced representation reduces model robustness and limits their translational potential in real-world NP discovery scenarios. Beyond structured databases, the broader scientific literature remains an untapped reservoir of chemical and biological information related to natural products. It is estimated that more than 10,000 chemistry-related articles are published annually, far exceeding the capacity for manual curation or systematic review [28]. As a result, valuable insights—especially those embedded in supplementary materials, technical reports, patents, or dissertations—often remain buried and inaccessible without the aid of advanced NLP tools. The challenges are further compounded when mining traditional or historical knowledge sources. Much of the information from ancient medical systems was transmitted orally or preserved in non-standardized textual formats, complicating efforts to digitize, verify, or compare such data across traditions [36]. These records often lack formal quality control or scientific validation, contributing to inconsistencies, ambiguities, and data gaps. Furthermore, the linguistic and cultural diversity inherent in traditional literature introduces a wide range of terminologies, metaphors, and context-specific meanings, which can obscure the interpretation and translation of claimed bioactivities. Overcoming these barriers requires the development of NLP frameworks specifically tailored to the unique characteristics of both contemporary scientific literature and traditional ethnomedical records. This includes designing ontologies that accommodate culturally diverse terminologies, integrating multilingual corpora, and applying machine translation models trained on domain-specific vocabularies [36]. Ultimately, advancing AI-driven NP discovery will depend on the creation of curated, standardized, and interoperable datasets enriched with both chemical and biological annotations. These datasets must include both positive and negative results, accurately represent stereochemistry, and be made openly accessible in machine readable formats to enable large-scale training, validation, and benchmarking. Without such infrastructure, the full promise of AI–NP synergy will remain unrealized.

**Table 2:** Data challenges in NP research and AI-enabled solutions

Data challenge	Description	Impact on NP based drug discovery	Strategic interventions	AI-enabled approaches
Unstructured and incomplete data	Many NP databases are inconsistently formatted, partially annotated, or rarely updated	Suboptimal for ML training; hampers reproducibility and integrative analysis	Promote data standardization protocols; incentivize comprehensive dataset curation across public repositories	ML for automated annotation; AI-assisted data imputation; use of ontologies and schemas to ensure interoperability
Buried or dispersed corpora	Critical data (e.g., in patents, dissertations, supplementary files) remain hidden in unindexed or inaccessible formats	Hinders access to untapped bioactivity and compound information, especially from non-standard sources	Develop open-access centralized repositories; support multilingual and culturally inclusive curation	NLP models for corpus mining; semantic search algorithms; AI-powered entity recognition across heterogeneous sources
Absent or ambiguous stereochemical data	Stereochemical details are frequently missing or ambiguously encoded in datasets, impairing modeling precision	Reduces the accuracy of docking, QSAR models, and pharmacophore predictions	Require stereochemical submission standards in NP databases and journals	ML-based stereochemistry prediction using cheminformatics tools (e.g., RDKit); quantum-informed AI for 3D inference
Underreporting of negative results	Negative or null bioactivity data are rarely published or shared, creating a biased data landscape	Leads to overfitting and unrealistic performance metrics in predictive modeling	Encourage journal editors and funders to mandate reporting of negative data; establish preprint sections for such results	NLP models to extract implicit negative findings from literature; AI to curate and rebalance datasets via synthetic negatives
Cultural context noise in ethnopharmacology	Traditional medical texts often include metaphorical language, regional idioms, and non-standard nomenclature	Impedes consistent interpretation, translation, and digital integration of ethnomedical knowledge	Collaborate with cultural experts; design ontologies reflecting indigenous and regional health paradigms	Culturally aware NLP frameworks; multilingual translation models trained on domain-specific corpora; contextual AI reasoning

### Legal and ethical constraints for commercialisations

While not rooted in chemical or biological properties, legal and ethical constraints—particularly around intellectual property (IP)—pose significant barriers to the commercial development of NP. Chief among these is the challenge of patentability. Because naturally occurring compounds are not considered inventions per se, they are often excluded from patent protection unless they are structurally modified, formulated in novel ways, or applied to previously unrecognized uses [37]. This lack of robust IP protection limits commercial incentives and deters investment in NP-based drug development.

Patent eligibility requires that an invention be novel, non-obvious, and useful. However, by definition, many NPs already exist in nature and may have long been known or used in traditional medicine systems. To meet the legal standard of novelty, researchers must frame their discoveries carefully, often focusing on previously uncharacterized compounds, new therapeutic mechanisms, or proprietary formulations. Typically, NP-related patents fall into one or more of the following categories: 1. Newly isolated natural compounds that have not been previously extracted, characterized, or described using any known technical method; 2. Novel therapeutic or cosmetic uses of known compounds based on previously unrecognized mechanisms of action or clinical indications; 3. Synergistic combinations of two or more natural products producing effects not previously described; 4. Innovative formulations, including optimized ratios, excipient blends, or delivery vehicles that enhance efficacy, stability, or bioavailability; 5. Advanced drug delivery systems or medical devices specifically designed to administer NP-based therapeutics with improved precision or safety.

Importantly, the interpretation and enforceability of these categories vary significantly across jurisdictions, complicating the global patenting landscape for NP innovations [37, 38]. Navigating this complex legal terrain can be time-consuming and uncertain, often discouraging academic researchers and industrial partners from pursuing commercialization pathways.

In parallel with legal constraints, ethical considerations—especially those involving biopiracy—pose additional challenges. Biopiracy refers to the unauthorized use, patenting, or commercial exploitation of biological materials or traditional knowledge without the consent or fair compensation of the originating communities. Accusations of biopiracy can arise when researchers or corporations derive products from indigenous knowledge systems without engaging in prior informed consent or equitable benefit-sharing agreements.

Several prominent biopiracy cases have shaped international discourse and influenced legal frameworks. In one well-known example, the European Patent Office granted a patent to W.R. Grace and the U.S. Department of Agriculture for a neem-based fungicidal formulation. The Indian government successfully contested this claim by providing documentation from classical Ayurvedic texts describing neem's traditional use, leading to the revocation of the patent [39].

A similar case occurred in 1995, when the University of Mississippi Medical Center obtained a U.S. patent on the wound-healing properties of turmeric. Indian authorities challenged the patent with references to traditional sources, ultimately resulting in its cancellation [40]. In another controversial example, French researchers patented Simalikalactone E, an anti-malarial compound derived from *Quassia amara*, based on ethnobotanical knowledge obtained from Indigenous Amazonian communities. Despite early benefit-sharing commitments, the patent raised concerns over restricted access for local populations and the potential loss of communal rights over medicinal plants [41].

These cases underscore the ethical tensions that arise when traditional knowledge is transformed into proprietary intellectual property. They have prompted widespread discussion on indigenous rights, cultural sovereignty, and the responsibilities of researchers operating in biodiversity-rich regions. Global policy instruments such as the CBD and the Nagoya Protocol have since been adopted to regulate access to genetic resources and to ensure fair and equitable benefit sharing.

As NP discovery becomes increasingly digitized, AI-driven, and global in scope, legal and ethical frameworks must evolve accordingly. Ensuring scientific progress while protecting the rights of knowledge holders and source communities will require ongoing collaboration between policymakers, legal experts, researchers, and Indigenous representatives. Robust regulatory safeguards, transparency, and culturally respectful partnerships will be essential to establishing a just and sustainable path forward for NP-based innovation.

## AI and machine learning in natural product discovery: tools reshaping the field

The integration of AI and ML into NP research has become a transformative force, reshaping how bioactive compounds are identified, optimized, and validated. These computational approaches are especially valuable in addressing the intrinsic complexity, structural diversity, and data limitations that have historically slowed NP-based drug discovery. By accelerating compound screening, predicting pharmacologically relevant properties, and enabling de novo molecular design, AI and ML are helping to unlock previously inaccessible regions of NP chemical space.

When combined with genomic, chemical, and pharmacological datasets, AI-driven methods can reveal hidden relationships, predict bioactivity profiles, and facilitate the rational design of NP-inspired drug candidates. This synergy is not only accelerating early-stage discovery but also enhancing the precision and scalability of NP-based research [35]. In this section, we provide an overview of key AI/ML methodologies currently being applied or adapted to address NP-specific challenges.

### Foundational algorithms in AI-driven NP research

AI and ML systems rely on a diverse array of algorithmic frameworks tailored to different stages of NP discovery. Among these, graph neural networks (GNNs), transformers, and variational autoencoders (VAEs) have shown particular utility in navigating the chemical and biological complexity of natural products.

#### Graph neural networks (GNNs):

GNNs represent molecules as graphs, where atoms are encoded as nodes and chemical bonds as edges. This format allows GNNs to effectively capture the intricate topologies found in natural products, synthetic scaffolds, and hybrid structures [28, 42]. These models have been successfully applied in a range of tasks, including the prediction of drug–target and drug–drug interactions, synthetic route planning, and de novo molecular design [43].

To address the interpretability limitations of traditional GNN models, recent efforts have introduced techniques such as Substructure Mask Explanation (SME). SME operates by segmenting molecules into chemically meaningful substructures and masking them individually to determine their relative contributions to model predictions. This approach offers insights akin to a chemist’s reasoning, enhancing trust in predictive outcomes and supporting structure–activity relationship (SAR) optimization [44]. Importantly, GNNs offer unique advantages for addressing NP-specific challenges. For stereochemical complexity, recent GNN architectures incorporate 3D coordinate encoding and chirality-aware featurization, enabling models to distinguish between enantiomers and diastereomers that exhibit dramatically different biological activities [45, 46]. For polypharmacology—a hallmark of many NPs—multi-task GNN architectures can simultaneously predict activity across multiple target panels, capturing the multi-target profiles characteristic of compounds like curcumin or quercetin [47]. Benchmark studies on NP-like molecules from the MoleculeNet dataset demonstrate that GNNs with explicit stereochemical encoding achieve higher accuracy on activity prediction tasks compared to 2D-only representations [46, 48]. The integration of GNNs with interpretability frameworks such as SME is poised to substantially accelerate lead optimization in NP-derived drug development.

#### Transformers

Originally developed for NLP, transformer models utilize self-attention mechanisms to capture long-range dependencies within sequences. In the context of molecular science, this architecture has been adapted to interpret both sequence- and structure-based chemical data, supporting tasks such as property prediction, large-scale virtual screening, and molecular structure translation [49].

Transformers form the backbone of major language models such as BERT and GPT and have been extended into cheminformatics, biochemistry, and materials science [50]. In NP research, they excel at recognizing subtle chemical and pharmacological patterns across complex datasets, improving performance in drug-likeness prediction, reaction outcome forecasting, and biological activity estimation. Their versatility in processing multiple modalities of data make them increasingly valuable in computational drug discovery. For natural products specifically, the self-attention mechanism of transformers proves particularly valuable for capturing long-range intramolecular interactions characteristic of macrocyclic NPs, where atoms separated by many bonds may interact spatially. Models such as MolBERT and ChemBERTa encode SMILES strings using sub-word tokenization, learning chemical semantics that enable SMILES-to-bioactivity prediction [51, 52]. Cross-modal transformers can jointly process chemical structure representations (SMILES or molecular graphs) alongside textual descriptions of biological activity, enabling extraction of structure–activity relationships from literature—a capability particularly relevant for mining ethnopharmacological texts where chemical and bioactivity information coexist [53, 54].

#### Variational autoencoders (VAEs)

VAEs are generative deep learning models designed to learn latent representations from high-dimensional data. In molecular applications, VAEs encode chemical structures into a continuous latent space, from which novel or related molecules can be generated [55]. Each model typically consists of an encoder, which compresses the input structure into latent variables, and a decoder, which reconstructs or generates molecules from this abstracted representation. In NP discovery, VAEs encode discrete chemical structures (e.g., graphs, fragments, or SMILES) into latent variables from which novel or closely related NP-like molecules can be decoded with high chemical validity.

A prominent example is the Natural Product-oriented Variational Autoencoder (NP-VAE), developed by Ochiai and colleagues, which was specifically trained to handle the size, branching, and stereochemical richness of natural product libraries [56]. NP-VAE combines fragment-based decomposition, tree-structured encodings, and ECFP-like features to construct a latent space that faithfully represents large, chiral NP scaffolds, achieving superior reconstruction accuracy and validity compared with earlier chemical VAEs. This architecture enables the generation of novel NP-like structures that maintain complex 3D frameworks while optimizing drug-relevant properties such as QED, synthetic accessibility, and filter-based ‘medicinal chemistry friendliness’ scores.

Although these algorithmic frameworks represent only a subset of the growing AI/ML toolkit in NP discovery, they illustrate the range and depth of current computational capabilities. A full accounting of AI methodologies applied to NP research is beyond the scope of this section and has been reviewed extensively elsewhere [35, 57]. Nevertheless, these examples highlight the transformative potential of AI in enabling efficient, scalable, and intelligent NP discovery pipelines—paving the way toward a new era of natural product-inspired therapeutics.

### AI-ready natural product databases and curation strategies

Reliable, well-curated databases are foundational to the development of high-performance AI and ML models in NP research. As discussed previously, the complexity and heterogeneity of NP data pose significant challenges for predictive modeling, dereplication, and virtual screening. However, several purpose-built databases have emerged in recent years, providing structured, annotated, and increasingly interoperable resources for AI-driven drug discovery. Here, we highlight three prominent databases—GNPS, NPASS, and COCONUT—that exemplify the integration of natural product data with modern informatics and curation strategies.

#### GNPS: global natural products social molecular networking

GNPS (Global Natural Products Social Molecular Networking) is a community-curated platform developed at the University of California, San Diego’s Center for Computational Mass Spectrometry. It provides an open-access environment for the deposition, analysis, and reanalysis of tandem mass spectrometry (MS/MS) data from natural product extracts [58]. The platform supports molecular networking to cluster spectra based on structural similarity, enabling automated dereplication and facilitating the annotation of previously uncharacterized compounds.

A key feature of GNPS is its dynamic, continuously updating spectral libraries. Uploaded datasets are automatically reanalyzed each month using newly integrated tools, allowing for the real-time annotation of novel or putative analogues. As of early 2021, GNPS had accumulated over 1,800 public datasets, 490,000 MS files, and more than 1.2 billion MS/MS spectra, attracting a global user base from over 160 countries [59]. The platform includes approximately 50 modular tools for data exploration, visualization, and interpretation, making it particularly useful for untargeted metabolomics workflows and AI-assisted annotation in NP discovery pipelines.

#### NPASS: natural product activity and species source database

NPASS (Natural Product Activity and Species Source Database) is a specialized resource that bridges chemical structure, biological activity, and organismal origin. The database was initially released with approximately 35,000 natural products from 25,000 species, linked to more than 5,800 biological targets and nearly half a million quantitative activity records (including IC₅₀, EC₅₀, Kᵢ, and GI₅₀ values) [60]. Recent updates to NPASS have expanded its coverage substantially. The database now includes over 95,000 additional records, 1,500 NP clusters, and approximately 400 new species entries. Significant enhancements include a 40% increase in NP bioactivity data, 32% growth in associated target annotations, and the addition of ADMET properties. NPASS also integrates tools such as Chemical Checker for chemical similarity assessment [61].

Crucially, NPASS complements structure-focused databases by offering richly annotated metadata, including MOA information and detailed taxonomical context. This depth of information enables the training of more predictive and context-aware AI models, particularly for target identification and lead prioritization.

#### COCONUT: collection of open natural products

COCONUT (Collection of Open Natural Products) is one of the most comprehensive general-purpose open-access NP databases to date. Developed by Sorokina et al. at Friedrich Schiller University, it aggregates data from over 50 sources, including ChEMBL, GNPS, NPASS, PubChem, and NP Atlas [62]. As of 2024, COCONUT contains more than 730,000 NP entries, with stereochemistry and taxonomy data included where available. The platform allows users to perform structure, similarity, and substructure searches, and supports bulk downloads in formats such as SDF, CSV, and SQL.

COCONUT 2.0 introduced a range of updates aligned with FAIR (Findable, Accessible, Interoperable, Reusable) data principles, including an improved interface, streamlined deposition workflows, and better tools for data reuse and reproducibility [63]. Its versatility has enabled a wide range of applications, including fragment-based scaffold identification, NP-likeness scoring, HTS design, and synthetic feasibility prediction.

Importantly, COCONUT also supports use cases beyond conventional cheminformatics. For example, the database has been employed in phenotype-driven drug discovery by linking traditional medicine records with disease-relevant phenotypes. It has also been used in target validation through compound–gene interaction analysis, and as a foundation for AI-enabled pipeline development, including the NaCTR framework, which supports NP-based drug discovery from traditional oriental medicine [64].

Together, GNPS, NPASS, and COCONUT form the backbone of a new data infrastructure for AI-powered natural product research. When paired with modern curation strategies—such as automated data extraction, standardized annotation protocols, and cheminformatics pipelines—these resources enable not only model training and validation but also the development of full-stack, end-to-end discovery platforms. As AI continues to evolve, the quality, accessibility, and interoperability of NP data will be critical to unlocking its full potential in therapeutic innovation.

### Cheminformatics and natural language processing: enabling technologies in natural product discovery

The convergence of cheminformatics, representation learning, and NLP is transforming the landscape of NP–based drug discovery. These technologies facilitate the translation of complex chemical structures and diverse textual knowledge into machine-readable formats, enabling AI models to extract patterns, predict activity, and generate novel molecular entities. Natural products, with their structurally rich and diverse scaffolds—including macrocycles, alkaloids, and polyketides—have traditionally challenged conventional computational tools. However, recent advances in chemical representation, bioinformatics, and cross-disciplinary informatics have made it increasingly feasible to model and analyze NP structures for combinatorial design, target prediction, and pharmacological profiling [65–67].

Early cheminformatics efforts to digitize NP chemical space emerged in the early 2000s, using dimensionality reduction techniques such as principal component analysis (PCA) and self-organizing maps (SOMs) to visualize high-dimensional molecular datasets [28, 68]. These approaches laid the groundwork for the adoption of ML classifiers in the following decade, which were applied to predict NP biological activity. More recently, deep neural networks have been employed in genome mining, structural annotation, and de novo molecular generation [69, 70].

#### Molecular representations and structural embeddings

A foundational concept in cheminformatics is the conversion of chemical structures into numerical embeddings that preserve molecular features and interrelationships. These vectorized representations form the basis for AI/ML models to learn complex SARs, assess drug-likeness, and predict pharmacological potential.

Two of the most widely used molecular representations are SMILES (Simplified Molecular Input Line Entry System), which encodes chemical structures as linear textual strings, and InChI (International Chemical Identifier), a standardized representation designed to improve interoperability and compound comparison [71, 72]. These formats serve as inputs for a wide range of deep learning architectures, including autoencoders, transformers, and graph neural networks, supporting tasks such as virtual screening, property prediction, and activity classification.

#### NLP for mining scientific literature and traditional knowledge

In parallel with cheminformatics, NLP—particularly transformer-based large language models (LLMs)—has emerged as a transformative tool for extracting meaning from the vast and growing corpus of scientific and traditional medical literature. BERT (Bidirectional Encoder Representations from Transformers), a foundational model developed by Google, was initially trained on general text corpora. Biomedical adaptations such as BioBERT and BioMed-RoBERTa extended this architecture to domain-specific literature, improving the extraction of entities and relationships from scientific texts [73–75]. Similarly, ChemBERTa, a model trained on chemical data including SMILES strings, has shown promise in molecular property prediction, similarity assessment, and virtual screening. Newer iterations such as ChemBERTa-77 M-MTR and ChemBERTa-2 offer improved performance for cheminformatics tasks [52]. These models are particularly valuable for mining large-scale databases of biomedical literature and patents, as well as for processing less-structured, historically rich sources such as traditional medicine compendia. The ability to link NPs to biological targets, diseases, or mechanisms of action directly from text is a major advantage for AI-powered discovery pipelines. The technical workflow for SMILES-to-bioactivity extraction involves multiple processing stages. First, chemical entity recognition identifies compound names and synonyms in text, which are then resolved to canonical SMILES using databases such as PubChem or ChEMBL. Second, bioactivity mentions (e.g., "inhibited," "EC50 = 10 nM") are extracted using named entity recognition fine-tuned on biomedical corpora. Third, relation extraction models link chemical entities to their associated activities and targets. Recent architectures employ cross-modal attention, where transformer encoders for SMILES representations attend to bioactivity text embeddings, enabling joint learning of chemical-semantic relationships [76]. This approach has demonstrated ~ 80% accuracy in extracting compound-target-activity triplets from pharmacological literature [77].

#### Applications in NP discovery and traditional knowledge integration

Although NLP and LLMs may appear distant from NP discovery at first glance, their application in mining traditional and ethnomedical texts is proving increasingly impactful. These sources, often unstructured, linguistically diverse, and lacking standardization, are difficult to process through manual curation alone. NLP offers a scalable solution to extract NP–disease associations, identify candidate compounds, and prioritize therapeutic leads based on centuries of accumulated empirical knowledge.

Recent advances underscore this potential. For example, Yang et al. developed a domain-specific NLP framework known as TCMDA (Traditional Chinese Medicine Domain Adaptation), which involved pretraining and fine-tuning LLMs on a 1-billion-token corpus of Traditional Chinese Medicine texts [78]. Their model, TCM-GPT-7B, outperformed general-purpose biomedical LLMs in tasks such as named entity recognition, relation extraction, and question answering, suggesting that similar approaches could be extended to other traditional systems, including Ayurveda, Siddha, and Unani. These applications reveal a powerful opportunity to merge cheminformatics with text-based knowledge extraction, effectively bridging structural and semantic domains in NP research. The resulting systems can facilitate compound identification, therapeutic hypothesis generation, and evidence synthesis across modern and traditional knowledge bases.

In summary, the integration of cheminformatics, structural embeddings, and NLP provides a critical foundation for the next generation of intelligent NP discovery pipelines. From learning chemical features to unlocking ethnopharmacological insights buried in historical literature, these tools dramatically expand the horizons of AI-enabled research. As digital infrastructure continues to mature, this convergence will play an increasingly central role in the development of nature-inspired therapeutics.

### Validation strategies for AI models in natural product research

Before AI- and ML-based predictions can be confidently applied in NP discovery, it is essential to assess their reliability, robustness, and biological relevance. While comprehensive guidelines for model evaluation have been reviewed extensively elsewhere [79], a contextual overview of validation frameworks is particularly critical for researchers working at the interface of computational and natural product science (Table 3).

**Table 3:** Key validation metrics for AI applications in natural product-based drug discovery

Task type	Metric name	Definition	Hypothetical use case in NP research
Model evaluation & generalization	Data partitioning	Dividing datasets into training, validation, and test sets to prevent overfitting and evaluate model robustness	Ensures models trained on NP spectral or text data can generalize to novel compounds or documents from external datasets (e.g., new plant extracts or manuscripts)
	Cross-validation (e.g., k-fold)	Data is split into k subsets; the model is trained on k − 1 folds and validated on the remaining fold. Repeated k times	Especially useful for small NP datasets (e.g., rare microbial metabolomes) to maximize training efficiency and improve reliability
	External Validation	Evaluation using independent datasets not seen during model development	Testing dereplication or target prediction tools on new NP libraries from different taxa or ecological regions.t
Classification	Accuracy	Proportion of correct predictions over total predictions	Basic metric for classifying bioactivity presence/absence of NP compounds from screening datasets
	Precision (Positive Predictive Value)	Proportion of true positives among predicted positives	Critical in NP dereplication to minimize false leads, ensuring predicted active compounds are indeed bioactive
	Recall (Sensitivity)	Proportion of true positives correctly identified	Important for early-stage NP hit identification to avoid missing active compounds
	F1 score	Harmonic mean of precision and recall	Balances precision and recall for class-imbalanced datasets common in NP bioassay results
	AUROC (Area Under ROC Curve)	Measures ability to distinguish between classes at various threshold settings	Evaluates model discrimination power in bioactivity classification tasks
	MCC (Matthews Correlation Coefficient)	Correlation coefficient between observed and predicted classifications, considering all confusion matrix terms	Robust metric in highly imbalanced NP datasets; less biased than accuracy alone
	AUPR (Area Under Precision-Recall Curve)	Highlights precision-recall trade-offs, especially under class imbalance	More informative than AUROC when actives are rare (e.g., < 10% in large NP libraries)
Regression	Mean squared error (MSE)	Average of squared differences between predicted and actual values. Penalizes larger errors	Used in predicting NP properties like IC₅₀ or binding affinity from molecular descriptors
	Mean absolute error (MAE),	Average of absolute differences between predicted and true values. Less sensitive to outliers than MSE	Useful in ADMET property prediction of NP scaffolds
	R^2^ (Coefficient of Determination)	Proportion of variance explained by the model. Ranges from 0 to 1	Applied in QSAR models predicting biological or physicochemical properties of NPs

#### Model selection and evaluation criteria

The selection of an appropriate AI or ML model depends on several factors, including the type of learning task (for example, classification versus regression), the quality and quantity of available data, and the required level of interpretability. A central concept in ML is generalization—the model’s ability to perform accurately on previously unseen data. To assess generalization, a typical workflow involves partitioning the available dataset into three components: a training set, used to fit the model; a validation set, used for tuning hyperparameters and tracking model performance during training; and a test set, reserved exclusively for final performance evaluation [34].

For classification tasks, model performance is often quantified using metrics such as accuracy, precision, recall, F1 score, area under the receiver operating characteristic curve (AUROC), and Matthews correlation coefficient (MCC). In cases where datasets are highly imbalanced—a common scenario in NP research—area under the precision-recall curve (AUPR) may offer more informative performance estimates [79, 80]. For regression problems, metrics such as mean squared error (MSE), mean absolute error (MAE), and the coefficient of determination (R^2^) are commonly used [81].

To ensure reproducibility and reduce the risk of overfitting, cross-validation strategies are widely adopted. In *k*-fold cross-validation, for example, the dataset is partitioned into *k* subsets (folds), and the model is trained and validated *k* times, each time using a different fold as the validation set. This iterative process provides a robust assessment of model stability and enables the selection of hyperparameters that generalize well across different subsets of the data. A model that performs consistently across both the test set and cross-validation folds is typically considered suitable for application to independent datasets.

#### Experimental validation and biological relevance

While computational validation is crucial, experimental verification remains the gold standard for assessing the biological relevance of AI-based predictions. However, such experimental follow-up is often underutilized in NP–AI research pipelines [79]. For instance, AI predictions of ligand–receptor interactions can be evaluated using molecular docking simulations and subsequently tested through in vitro binding assays [82]. Similarly, predicted bioactivity values can be benchmarked against wet-lab assay results when such data are available.

Efforts to improve biological relevance have focused on integrating experimental datasets into model training pipelines, harmonizing assay conditions across public databases, and refining molecular representations to better reflect biological behavior. These strategies aim to bridge the translational gap between in silico predictions and empirical outcomes—an especially important step when working with the structural complexity and polypharmacology characteristic of many NPs.

#### Relevance for NP-focused researchers

Understanding model validation is no longer solely the purview of computational scientists. As AI becomes increasingly embedded in the workflow of NP discovery, researchers must be equipped to critically assess model quality, interpret performance metrics, and collaborate effectively with data scientists. This includes selecting appropriate validation frameworks, evaluating biological plausibility, and translating predictions into testable hypotheses.

Such interdisciplinary fluency enhances the credibility and applicability of AI-driven discoveries and helps ensure that computational insights lead to real-world therapeutic innovations. In the context of NP research, where compound availability and experimental throughput are often limited, the use of well-validated models becomes not just beneficial—but essential—for prioritizing leads and accelerating translational outcomes.

### AI-guided genome mining for cryptic biosynthetic pathways

A significant frontier in NP discovery lies in the genomic "dark matter"—the vast majority of biosynthetic gene clusters (BGCs) that remain unexpressed or poorly characterized under standard laboratory conditions. AI and machine learning are now enabling systematic mining of these cryptic pathways, dramatically expanding the accessible NP chemical space. antiSMASH (antibiotics & Secondary Metabolite Analysis Shell) represents the foundational platform in this domain, using rule-based and machine learning approaches to identify and annotate BGCs across bacterial, fungal, and plant genomes [83]. The associated antiSMASH-DB now contains over 200,000 predicted BGC regions from publicly available genomes, providing training data for more advanced ML approaches [84, 85]. DeepBGC extends these capabilities using bidirectional long short-term memory (BiLSTM) networks trained on Pfam domain sequences, achieving improved detection of novel BGC classes not captured by rule-based methods [86]. The model demonstrates particular strength in identifying hybrid BGCs—clusters combining biosynthetic logic from multiple pathways—which often produce structurally novel NPs.

PRISM (PRediction Informatics for Secondary Metabolomes) takes a complementary approach, using hidden Markov models and chemical logic to predict not just BGC presence but the likely chemical structures of encoded products [87]. This structure prediction capability enables prioritization of cryptic clusters likely to produce compounds with desired structural features. Recent work has applied transformer architectures to BGC-to-structure prediction, learning the "grammar" of biosynthetic assembly lines to generate candidate structures from genomic sequence alone [88, 89]. While still early-stage, these approaches promise to close the gap between genomic potential and chemical reality in NP discovery.

### AI for retrosynthetic planning of complex natural products

The structural complexity of many NPs—featuring multiple stereocenters, fused ring systems, and labile functional groups—poses formidable challenges for chemical synthesis. AI-driven retrosynthesis tools are increasingly capable of addressing these challenges, proposing viable synthetic routes to complex NP targets.

ASKCOS (MIT) and RetroPath (EMBL) represent leading platforms, using template-based and template-free approaches respectively to decompose target structures into achievable synthetic steps [90, 91]. For NPs, these tools must accommodate several domain-specific challenges: (1) preserving stereochemistry throughout multi-step sequences, (2) incorporating biocatalytic transformations that complement traditional organic chemistry, and (3) identifying protecting group strategies for polyfunctional intermediates. Molecule Chef and related generative models approach retrosynthesis as a sequence-to-sequence translation problem, learning to "translate" target structures into synthetic precursors [92]. When trained on datasets enriched with NP syntheses, these models demonstrate improved performance on complex terpene and alkaloid targets.

A particularly promising direction involves hybrid biosynthetic-chemical retrosynthesis, where AI systems propose routes combining fermentation-derived intermediates with chemical modifications [93]. This approach leverages the stereochemical precision of enzymatic transformations while accessing chemical space beyond natural biosynthetic logic. Such hybrid routes have proven valuable for semi-synthetic production of complex NPs like paclitaxel and artemisinin derivatives.

## Artificial intelligence in natural product discovery: transforming the pipeline from source to lead

NP–based drug discovery has long relied on phenotypic strategies rooted in ethnobotanical exploration. This process often begins with the examination of ancient medical texts, ethnopharmacological surveys, or the documentation of traditional healing practices through engagement with Indigenous and local knowledge systems. Once a promising bioactive source is identified, researchers initiate a discovery pipeline that integrates successive layers of chemistry, biology, and analytics, ultimately aimed at isolating and validating therapeutic compounds.

At the experimental level, the workflow typically begins with optimized extraction techniques—such as ultrasound-assisted extraction (UAE), microwave-assisted extraction, or hybrid solvent systems—designed to maximize yield and preserve bioactivity. Crude extracts are then subjected to bioassay-guided fractionation, supported by high-throughput in vitro screening. These workflows leverage advanced chromatographic platforms, including high-performance liquid chromatography (HPLC), flash chromatography, and preparative HPLC, often coupled with tandem mass spectrometry (MS/MS) for untargeted metabolomics. The resulting data provide a detailed metabolic fingerprint of each bioactive extract or fraction.

Metabolomic and bioassay data are then integrated for compound prioritization and bioactive metabolite deconvolution. This step increasingly relies on molecular networking approaches—such as those implemented via the GNPS (Global Natural Products Social Molecular Networking) platform—which allow researchers to cluster structurally related metabolites based on spectral similarity, correlate them with bioactivity patterns, and prioritize candidates for further investigation.

While this conventional workflow has enabled the discovery of numerous therapeutics, it also generates a staggering volume of complex, multidimensional data. From unstructured ethnopharmacological literature to high-resolution metabolomic spectra, the scale and heterogeneity of information routinely exceed the capacity of human analysis alone. This is precisely where artificial intelligence becomes transformative.

AI technologies offer powerful solutions to the data complexity, scale, and pattern-recognition challenges that characterize modern NP research. From automated mining of traditional medical texts and scientific literature, to compound clustering, activity prediction, SAR modeling, and de novo compound design, AI can be integrated at every stage of the NP discovery pipeline. These tools enable intelligent data curation, accelerate hypothesis generation, and support decision-making processes that would be otherwise infeasible using conventional methods.

In the following sections, we examine key applications of AI across the NP drug discovery continuum. We also propose a comprehensive, AI-integrated workflow that illustrates how computational technologies can be embedded at each stage—from initial source identification to lead optimization—offering a scalable and systematic framework for the future of natural product–based therapeutics.

### AI-powered text mining of traditional knowledge systems

As outlined earlier, NLP—a core subfield of AI—has become an indispensable tool for NP–based drug discovery. NLP enables computational models to process and extract meaning from human language, allowing researchers to mine structured insights from unstructured sources such as ancient medical texts, ethnobotanical records, and scientific literature (Table 4).

**Table 4:** Applications of NLP and LLMs in mining traditional medical literature

Study/tool	Data source	Model type / capability	Key outcomes	Refs
BioNLP (General)	Biomedical and pharmacological literature	Classical NLP pipeline	Enabled extraction of pharmacological terms, disease associations, and treatment relationships from semi-structured and unstructured biomedical corpora	[95]
May et al. (2014)	Ancient Chinese texts — Zhong Hua Yi Dian and Zhong Yi Fang Ji Da Ci Dian	Rule-based NLP and early ML	Developed an algorithm for entity recognition, classification, and scoring of therapeutic patterns. Enabled diachronic analysis of terminology evolution and common prescription tracking in TCM records	[98]
Qibo	Two-stage training approach with a 2 GB dedicated TCM corpus	Qibo based on Chinese-LLaMA in two stages from pre-training to SFT (supervised fine-tuning)	63% mean subjective win rate, improved mean objective accuracy from 23 to 58%	[101]
BioBERT, RoBERTa, etc	TCM digital libraries, biomedical corpora	Transformer-based models (100 M–125 M parameters)	Applied for TCM terminology normalization, named entity recognition (NER), relationship extraction, and detection of adverse drug reactions (ADRs) across multilingual and heterogenous sources	[74, 110]
GPT-4	Full-text TCM databases, historical prescriptions, clinical reports	Generative transformer model (175B–1800B parameters)	Used for prescription generation, literature summarization, and automated extraction of drug-effect relationships. Also applied for semantic matching and question-answering within ancient medical literature	[100]
LLaMA2 (7B–13B)	Digitized ancient TCM manuscripts, diagnostic dialogues, and formula compendia	Fine-tuned open-source LLM	Adapted for TCM-specific diagnostic tasks, multi-label text classification, and multilingual information retrieval. Enabled improved entity linking in noisy, culturally nuanced textual datasets	[100]

In this context, **BioNLP**—the adaptation of NLP methods to biomedical and pharmacological data—has emerged as a particularly powerful approach for unlocking historical and traditional knowledge systems. Disciplines such as Traditional Chinese Medicine (TCM), Ayurveda, Siddha, and Unani contain vast, underexplored repositories of therapeutic knowledge. AI-assisted mining of these texts offers a path toward identifying plant-based compounds with bioactive potential, many of which may hold clinical relevance for modern diseases [94, 95].

#### Early applications in TCM literature mining

Interest in applying AI to traditional medicine has grown significantly, especially following high-profile NP discoveries in areas such as antimalarial and anti-inflammatory therapeutics [96, 97]. One of the earliest efforts in this space was conducted by May et al. (2014), who developed algorithms to extract, classify, and score medicinal information from ancient Chinese medical literature [98]. Their system enabled the tracking of terminology evolution, identification of commonly used herbal formulations, and digital mapping of therapeutic patterns across centuries. These insights were drawn from sources such as the *Zhong Hua Yi Dian* and *Zhong Yi Fang Ji Da Ci Dian* (Great Compendium of Chinese Medical Formulae).

In a related study, Shergis et al. (2015) applied text mining techniques to identify 331 compounds—including those derived from herbs, minerals, and animal sources—as potential treatments for chronic cough, again using structured queries on classical TCM corpora [99].

#### Emerging role of LLMs

Recent advances in LLMs, including BERT and GPT architectures, have further expanded the scope of NLP in traditional medicine [100]. These models excel at interpreting large, complex textual datasets and have demonstrated utility in biomedical domains. For example, Zhang et al. (2024) developed Qibo, a LLM specifically tailored for TCM, using a two-stage training approach with a 2 GB dedicated TCM corpus [101]. Qibo demonstrates significant performance improvements in TCM NLP tasks and supports applications such as TCM consultation, addressing challenges posed by the theoretical differences between TCM and modern medicine.

LLM-based approaches are increasingly integrated into knowledge graphs, enabling the creation of semantic links among medicinal herbs, active ingredients, targets, and diseases [102]. Notably, platforms such as TCMBank now include over 9,000 herbs, nearly 62,000 unique ingredients, more than 15,000 biological targets, and 32,000 disease associations, with comprehensive pairwise relationships mapped between these entities [103].

#### LLM-powered tools and multimodal integration

The adaptation of LLMs for specialized interfaces has also led to new AI-powered platforms for real-time literature exploration. For example, InsilicoGPT provides a question–answering system that links user queries directly to relevant scientific paragraphs and references, facilitating rapid hypothesis generation and contextual understanding [104, 105].

The emergence of multimodal LLMs, capable of simultaneously processing text, images, audio, and even video, further enhances this potential. These models can synthesize information from heterogeneous sources—ranging from medical manuscripts and spectral data to ethnographic interviews—enabling tasks such as multi-step research question answering, document summarization, cross-modal annotation, and hypothesis generation [106]. This is particularly advantageous for NP researchers who frequently work with semi-structured, multilingual, and multimodal datasets.

#### Model selection and remaining challenges

Despite these advances, model selection remains a critical consideration. The “no free lunch” theorem reminds us that no single algorithm performs optimally across all tasks [107]. Choosing an appropriate LLM architecture depends on factors such as dataset size, domain specificity, and the desired output (e.g., entity extraction, relationship mapping, or question answering).

Several limitations still hinder broader adoption of LLMs in traditional knowledge mining. These include inconsistent terminologies across historical texts, non-standard data formats, limited access to digitized corpora, high computational overheads, and challenges with model tuning, alignment, and precision in domain-specific contexts [28, 108, 109].

#### Future directions

To realize the full potential of AI in traditional knowledge mining, future research should focus on several strategic directions. First, multimodal data integration—combining text, imagery, and audio—can improve the contextual understanding of traditional manuscripts and oral knowledge. Second, automated data labeling methods, such as weak supervision and active learning, can alleviate the bottleneck of manual annotation. Third, improvements in algorithmic efficiency, including lightweight LLM architectures optimized for biomedical and ethnopharmacological tasks, will reduce computational barriers. Finally, the standardization and expansion of machine-readable corpora, particularly in underrepresented systems like Ayurveda and Siddha, will enhance cross-cultural and comparative analysis.

By embedding these AI and NLP technologies into NP workflows, researchers can transcend human limitations in processing traditional knowledge and accelerate the discovery of therapeutics rooted in centuries of empirical wisdom.

### Dereplication 2.0: AI-driven acceleration from metabolomics to molecular leads

The emergence of "Dereplication 2.0" represents a paradigm shift in NP discovery—transforming a traditionally manual, time-intensive process into a data-driven, high-throughput, and AI-augmented step in the drug discovery pipeline. Dereplication, classically defined as the early identification of known compounds in complex extracts, serves as a critical checkpoint for avoiding the redundant re-isolation of previously characterized molecules. With the integration of ML and AI, dereplication has become not only more efficient and scalable, but also more informative, enabling early-stage filtering, spectral-based annotation, and structure prioritization [30, 111] ***(***Table 5***)***.

**Table 5:** AI tools for dereplication based on NMR and MS data

Tool name	Spectral modality (NMR/MS)	Model type / AI method	Key outcomes	Refs
COLMAR	NMR	computer-assisted structure elucidation (CASE)	Enables semi-automated annotation of metabolites in complex NMR datasets; supports comparison with NMR databases for dereplication of known metabolites in biological samples	[114, 115]
SMART-Miner				
shiftML	NMR	machine learning method based on local environments to accurately predict chemical shifts of molecular solids and their polymorphs to within DFT accuracy	Predicts solid-state NMR shifts with DFT-level accuracy using chemical graph information; successfully applied to resolve structures such as cocaine and complex drug scaffolds	[116]
SMART 2.0	NMR	Deep CNN (Convolutional Neural Network)	Trained on ~ 53,000 2D NMR spectra; projects spectra into a 180-dimensional latent space to support rapid dereplication and discovery of novel compounds like symplocolide A	[117]
DP4-AI	NMR	Bayesian inference + Automated probabilistic assignment	Automates the interpretation of^13^C and ^1^H NMR spectra for structure elucidation; accelerates structure assignment using Gaussian mixture models and theoretical shift matching	[118]
MetFID	MS	Artificial Neural Networks (ANN)	Outperformed conventional methods in ranking putative metabolite IDs, achieving top-rank accuracy in ~ 50% of test cases. Demonstrated superior performance over ChemDistiller, MetFrag, and CSI:FingerID	[125]
DeepEI	MS	Deep Neural Networks	Achieved > 90% accuracy and F1 scores above 79% for fingerprint prediction; demonstrated high recall in compound identification using electron ionization (EI) spectra	[126]
GNPS/ GNPS2	MS	Spectral similarity scoring, community molecular networking, clustering	Supports global-scale dereplication via cosine scoring and molecular network visualization; GNPS2 offers enhanced modular workflows, improved reproducibility, and real-time collaborative data curation for both academia and industry	[58]

#### AI-enhanced interpretation of NMR spectroscopy

NMR spectroscopy remains a cornerstone of NP structural elucidation. However, conventional interpretation is often limited by challenges associated with stereochemical complexity, conformational diversity, and low signal resolution in mixtures. AI-augmented tools are now addressing these limitations by predicting high-accuracy NMR chemical shifts, ranking candidate structures probabilistically, and reducing the risk of misassignments [112, 113].

Notable NMR tools include COLMAR and SMART-Miner, both computer-assisted structure elucidation (CASE) platforms designed to annotate metabolites in complex NMR datasets [114, 115]. The model shiftML, trained on GIPAW Density Functional Theory (DFT)-calculated shifts from the Cambridge Structural Database, enables accurate prediction of solid-state NMR shifts across diverse compound classes [116]. SMART 2.0, a convolutional neural network trained on more than 53,000 2D-NMR spectra, embeds spectra into a 180-dimensional latent space, facilitating dereplication and aiding in the identification of novel NPs such as *symplocolide A* [117].

Another advancement, DP4-AI, combines quantum chemical NMR shift predictions with Bayesian inference, assigning confidence scores to candidate structures [118]. Recent advances in quantum mechanical calculations integrated with machine learning have further enhanced structural elucidation capabilities. GFN2-xTB provides semi-empirical quantum mechanical calculations at dramatically reduced computational cost, enabling conformational sampling of large NPs that would be prohibitive with traditional DFT methods [119]. When combined with machine learning for NMR shift prediction (GFN2NMR), this approach achieves near-DFT accuracy at a fraction of the computational expense [120].

DU8ML (Deep Ultrafast 8-parameter Machine Learning) represents a more specialized approach, using neural networks to predict NMR parameters directly from 3D coordinates without explicit quantum mechanical calculations [121]. Trained on DFT-calculated parameters for diverse organic molecules, DU8ML enables rapid structure verification for putative NP structures generated during dereplication workflows.

#### AI applications in mass spectrometry–based dereplication

Mass spectrometry (MS), particularly tandem MS (MS/MS), produces dense, information-rich spectra ideal for AI-powered interpretation. Historically, MS-based structure prediction relied on rule-based methods dating back to the 1960s [35, 122]. Recent developments have embraced deep learning (DL) to interpret fragmentation patterns, predict molecular formulas, and even generate de novo candidate structures [123, 124].

For example, MetFID employs artificial neural networks trained on MS/MS spectra and molecular fingerprints to improve annotation accuracy [125]. DeepEI, another DL framework, aligns predicted and experimental fingerprints to match compounds in electron ionization (EI) spectra [126]. Community-based platforms such as GNPS (Global Natural Products Social Molecular Networking) leverage molecular networking to cluster related MS/MS spectra, streamlining dereplication and aiding structural classification [58].

#### The evolution to GNPS2: scalable, modular, and AI-integrated

The recent launch of *GNPS2*, developed by the Wang Bioinformatics Group at UC Riverside, marks a significant advancement in MS-based dereplication. As of July 2025, GNPS2 hosts over 2.4 million MS/MS spectra and features real-time spectral library updates, enhanced user support, and a modular workflow engine for reproducibility. The platform is rapidly becoming a central hub for AI-integrated NP dereplication and is accessible at https://gnps2.org [127].

#### Advanced tools and structural class inference

Several specialized tools now complement GNPS2 in the dereplication ecosystem. CSI:FingerID, along with its derivatives such as SIRIUS 4, MS2LDA, VarQuest, and DEREPLICATOR + , applies support vector machines, kernel learning, and fragmentation tree inference to match MS/MS spectra with structural databases [122, 123, 128, 129] [130]. In parallel, *CANOPUS*, a deep learning tool, enables classification of unknown compound classes directly from MS/MS data—even in the absence of known structural analogues—providing insights into the chemical novelty of detected compounds [131].

#### High-throughput libraries and MSⁿ-enabled learning

One historical bottleneck in dereplication has been the scarcity of high-quality, comprehensive spectral libraries. This limitation is being addressed through innovations in multi-stage fragmentation (MSⁿ) and automated spectral curation. *MSnLib* represents a significant step forward, comprising over 1.1 million MSⁿ spectra from 16,000 unique compounds—acquired in just 12 days of data collection. The platform enables substructure matching, de novo fragment interpretation, and generation of training sets for machine learning models focused on fragmentation–substructure relationships [132]. MSnLib also integrates with tools like MZmine, ensuring interoperability, quality control, and reproducibility in downstream workflows.

#### Open access, scalability, and the future of dereplication

The expansion of open-access resources such as GNPS2 and MSnLib marks a critical inflection point in NP-based dereplication. These repositories not only serve as training sets for AI models and validation frameworks for structural prediction but also promote transparent, community-driven discovery workflows. The integration of AI-powered dereplication tools with classical NP pipelines can significantly reduce redundancy, enhance chemical novelty, and accelerate the identification of promising leads.

In summary, *Dereplication 2.0* transforms a historically labor-intensive, low-throughput process into a fast, intelligent, and scalable operation. By embedding spectral intelligence within advanced algorithms and open infrastructures, researchers are now equipped to navigate the vast chemical space of natural products with significantly accelerated timelines and precision—ultimately shortening the path from extract to novel therapeutic lead.

## Case studies in action: realizing AI’s promise in natural product discovery

The integration of AI and ML has begun to fundamentally reshape NP drug discovery, yielding tangible advancement across diverse therapeutic areas. From antimicrobial resistance to oncology and the modernization of traditional medicine systems, AI has accelerated multiple stages of the drug development pipeline—streamlining lead identification, elucidating mechanisms of action, and expanding access to previously untapped chemical space. This section highlights select case studies that demonstrate the real-world impact of AI-enabled natural product discovery and provides key insights into success factors and remaining challenges (Table 6).

**Table 6:** AI-driven antibiotic and anticancer drug discovery: summary of key studies

Compound / NP	Target pathogen / cancer type	Biological target	AI method used	Outcome	Refs
Halicin	Escherichia coli, Mycobacterium tuberculosis, carbapenem-resistant strains	Bacterial respiration and membrane potential	Deep neural networks trained on ~ 2,300 compounds from Drug Repurposing Hub	Identified a novel, broad-spectrum antibiotic structurally distinct from known classes; validated in vitro and in vivo	[134]
Abaucin	Acinetobacter baumannii	Not target-specific (narrow-spectrum profiling)	DL-based screening with customized target pathogen dataset	Discovered a narrow-spectrum antibiotic with minimal off-target activity; precision-targeted pathogen inhibition	[133]
Mammuthusin-2 / Elephasin-2	Multidrug-resistant Gram-negative pathogens	Cytoplasmic membrane depolarization	Generative deep learning (APEX model), trained on ancient proteomic datasets	De-extinct peptides from extinct species with efficacy comparable to polymyxin B; active in preclinical mouse models	[135]
Paclitaxel (Taxol)	Non-small cell lung cancer	Microtubules	Neuro-fuzzy inference systems, genetic algorithms, reinforcement learning	Optimized biosynthetic yields and dose prediction; improved pathway modeling and metabolic engineering	[138–140]
Bruceine D / Eleutherobin / PMA	Various cancers (e.g., leukemia, colon)	Microtubules, inflammation pathways	Deep learning for scaffold screening and mechanism-of-action prediction	Identified novel NP-based microtubule inhibitors with potential anticancer properties	[141]
Scutellaria-derived NP	Various cancers (PLK1-overexpressing tumors)	Polo-like kinase 1 (PLK1)	Hybrid experimental-AI pipeline with covalent docking and target-site analysis	Discovered covalent PLK1 inhibitor targeting cysteine residues; validated binding and activity	[142]
Marinopyrrole A analogs	Inflammatory-driven cancers	Cyclooxygenase-1 (COX-1)	AI-based de novo analog design and docking screening	Generated analogs with ~ 160 × improved potency (IC₅₀ in nanomolar range) over the parent NP	[143]

### Antibiotic discovery: halicin, abaucin, and the dawn of molecular de-extinction

Antibiotic discovery, long hampered by diminishing returns and rising resistance, has been reinvigorated by the application of AI [133]. Notably, deep learning models are now enabling researchers to screen vast chemical libraries with accelerated timelines and accuracy—identifying structurally novel compounds that fall outside conventional scaffolds.

A landmark example is *Halicin*, a broad-spectrum antibiotic identified by researchers at MIT using a deep neural network trained on a dataset of 2,335 compounds with known *E. coli* inhibition profiles. The model screened over 100 million compounds from the Drug Repurposing Hub in just days—a feat unattainable by traditional methods. Halicin not only exhibited potent activity against *E. coli*, carbapenem-resistant *Enterobacteriaceae*, and *Mycobacterium tuberculosis*, but also displayed structural novelty, reducing the likelihood of resistance due to class cross-reactivity. Importantly, 23 additional active compounds were predicted by the same model, including eight with novel scaffolds [43, 134].

Building on this success, Liu et al. used a similar deep learning strategy to identify *Abaucin*, a narrow-spectrum antibiotic effective against *Acinetobacter baumannii*, a notoriously resistant Gram-negative pathogen. Unlike Halicin, Abaucin showed highly selective antimicrobial activity, highlighting AI’s ability to identify compounds tailored to specific pathogens—minimizing collateral damage to the host microbiome [133].

An even more audacious application of AI in antibiotic discovery is APEX (Antibiotic Peptide De-extinction)—a model that mines extinct proteomes to uncover lost antimicrobial peptides (AMPs). Trained on over 100 million peptide sequences, APEX identified ~ 37,000 putative AMPs, including ~ 11,000 with no analogues in living species. Among these were *mammuthusin-2* (from *Mammuthus primigenius*) and *elephasin-2* (from *Elephas antiquus*), both of which showed potent efficacy in preclinical models [135]. These extinct-derived AMPs employed novel mechanisms, such as cytoplasmic membrane depolarization, offering new strategies to circumvent existing resistance pathways.

These case studies underscore the transformative role of AI in reviving antibiotic pipelines—cutting costs, increasing precision, and unearthing novel molecular scaffolds that would otherwise remain hidden.

### Anticancer discovery: AI-guided derivatization and targeted innovation

The application of AI and ML is rapidly transforming the landscape of anticancer drug discovery, particularly in the context of structurally complex NP. Historically, the development of NP-derived anticancer agents has relied heavily on empirical screening and trial-and-error structural derivatization—processes often constrained by limited availability of source material, synthetic inaccessibility, and suboptimal pharmacokinetics [136, 137]. AI now offers promising solutions to these challenges, enabling rational biosynthetic modeling, mechanism-specific molecular design, and de novo generation of NP-inspired analogs with enhanced drug-like properties.

A prominent case study in this context is *paclitaxel (Taxol)*, a chemotherapeutic initially isolated from the bark of *Taxus brevifolia*. While its discovery in the 1960s was driven by classical bioactivity-guided screening, recent AI-enabled approaches have significantly improved both its production and therapeutic precision. For instance, a hybrid system combining adaptive neuro-fuzzy inference systems (ANFIS) with genetic algorithms (GA) has been used to optimize culture conditions for paclitaxel biosynthesis in *Corylus avellana* cell cultures—outperforming conventional statistical models in yield prediction and culture scaling [138]. At the clinical interface, reinforcement learning algorithms have been applied to simulate precision dosing strategies in non-small cell lung cancer patients, using Bayesian data assimilation to balance efficacy with toxicity, thus refining real-world treatment protocols [139].

Further upstream in the production pipeline, AI has proven effective in addressing gaps in the paclitaxel biosynthetic pathway. Genome mining and AI-guided metabolic engineering have helped elucidate missing or poorly characterized enzymes, paving the way for scalable semi-synthetic and bio-manufacturing routes that reduce dependency on rare natural sources [140]. AI is also enabling the identification of novel NP-based microtubule inhibitors that go beyond classical scaffolds such as paclitaxel. Deep learning models have been used to identify compounds such as bruceine D, eleutherobin, and phorbol 12-myristate 13-acetate, each of which exhibits potent microtubule-disrupting activity derived from distinct natural frameworks [141]. These findings highlight AI’s ability to prioritize structurally diverse compounds based on mechanism-of-action alignment with validated oncology targets.

A particularly compelling example of target-specific NP repurposing comes from the work of Hao Liang and colleagues, who developed a hybrid AI–experimental pipeline to discover covalent inhibitors of polo-like kinase 1 (PLK1), a mitotic regulator overexpressed in multiple cancers. By focusing on NPs predicted to covalently bind cysteine residues (Cys67 and Cys133) in PLK1’s ATP-binding pocket, the team identified a compound from *Scutellaria baicalensis* that was experimentally validated as a highly specific PLK1 modulator [142].

Beyond repurposing, AI is also driving de novo NP-inspired molecular design. One illustrative case involves marinopyrrole A, a marine-derived NP from *Streptomyces* species. Using AI-guided structure generation algorithms, researchers designed a suite of COX-1 inhibitor analogs with markedly improved potency. The most effective compound demonstrated an IC₅₀ of 0.101 ± 0.051 µM—representing more than a 100-fold enhancement over the parent molecule (IC₅₀ = 16.6 ± 2.3 µM) [143]. This case exemplifies AI’s capacity to distill pharmacologically relevant features from complex NP scaffolds and reconstruct them into synthetically tractable analogs with optimized efficacy and safety profiles. Refer Supplementary file (Fig S1) containing the 2D chemical structures of key compounds.

### Modernizing traditional medicine: AI at the interface of ethnopharmacology and precision therapeutics

AI is playing an increasingly central role in the modernization of traditional medical systems—particularly in transforming NP from ethnobotanical knowledge into precision therapeutics. This paradigm shift is especially evident in Traditional Chinese Medicine (TCM), which has served as a model framework for integrating AI into multi-component, multi-target therapeutic strategies. Unlike conventional drug discovery approaches that typically focus on single-target agents, TCM is characterized by its use of complex herbal formulas composed of multiple bioactive constituents designed to act synergistically across diverse biological pathways [108, 144, 145]. This holistic pharmacological philosophy aligns well with modern systems biology and network pharmacology approaches, making it fertile ground for AI-driven analysis [146].

Over the past decade, a growing ecosystem of specialized TCM databases has emerged, providing structured access to traditional prescriptions, phytochemical profiles, disease indications, and predicted molecular targets. Notable resources include SymMap[147], YaTCM [148], TCMSP [149], TCMID [150], SuperTCM [151], ITCM [152], HIT 2.0 [153], TM-MC 2.0 [154], BATMAN-TCM 2.0 [155], TCM-suite [156], and TCM Bank [103]. These platforms serve as foundational infrastructure for AI applications in TCM research.

For example, *SymMap* maps relationships between botanical agents, TCM-specific symptomatology, and modern disease classifications, facilitating cross-cultural pharmacological inference. *TCMSP* compiles comprehensive ADME (absorption, distribution, metabolism, and excretion) profiles for commonly used TCM metabolites, supporting predictive modeling of drug-likeness and bioavailability. *YaTCM* integrates pathway information and protein target data with classical TCM formulas, enabling AI models to predict potential mechanisms of action. *TCM-suite* goes a step further by combining multi-omics datasets, phytochemical fingerprinting, and network pharmacology algorithms into a cohesive platform for hypothesis generation and lead prioritization.

Meanwhile, *TCM Bank*, one of the most advanced resources, applies big data analytics and unsupervised learning to identify adverse drug reactions and predict drug–disease interactions, thereby facilitating model generalization and real-world clinical applicability. These resources have collectively laid the groundwork for the transformation of TCM into a more evidence-based, precision-guided discipline, where ancient wisdom is recontextualized using modern computational techniques.

**Fig. 3:**
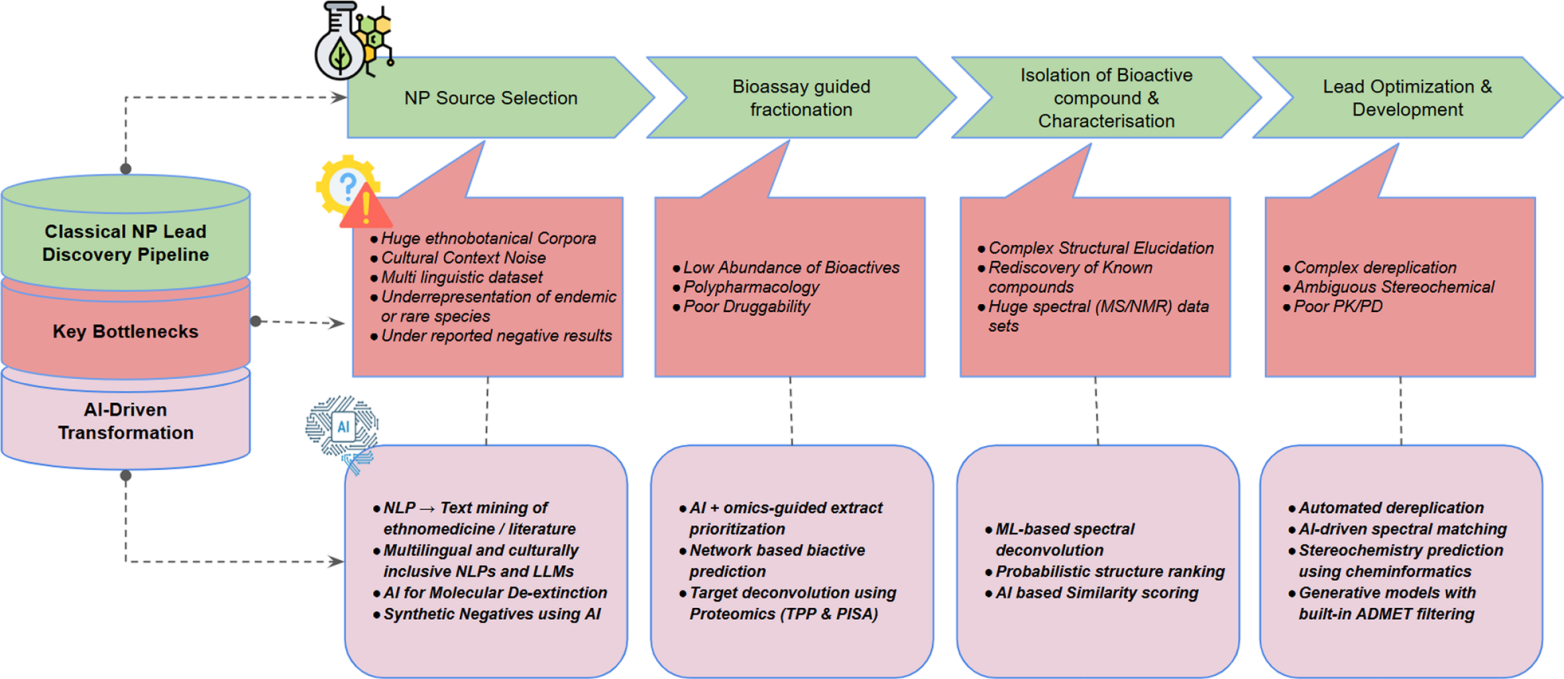
AI-augmented roadmap for natural product drug discovery: from bottlenecks to transformation. This schematic illustrates the classical NP–based lead discovery pipeline, highlighting critical bottlenecks at each stage—from NP source selection to lead optimization. AI-driven innovations are shown addressing Key limitations through text mining of traditional knowledge, omics-guided extract prioritization, machine learning–based spectral deconvolution, SAR modeling, and generative compound design. Together, these tools offer a scalable, efficient, and inclusive framework to revitalize NP-based drug discovery

The broader implication is clear: the modernization of traditional medicine via AI is not only feasible but essential. As similar frameworks are adapted for systems such as Ayurveda, Siddha, and Unani, a new era of globally integrated, AI-informed natural product discovery is rapidly taking shape ***(***Fig. 3***)***.

#### Lessons and outlook

These case studies—from antibiotics and anticancer agents to the modernization of ethnopharmacology—illustrate AI’s capacity to transform every phase of natural product discovery. Whether identifying ancient peptides from extinct species, optimizing NP biosynthesis, or translating traditional medicine into modern therapeutics, AI is redefining the tempo and trajectory of drug development.

Crucially, these examples also point to key success factors: access to curated data, cross-disciplinary collaboration, robust validation strategies, and transparency in AI workflows. While full regulatory approval of an AI-designed NP therapeutic remains on the horizon, the growing body of evidence suggests it is not a question of *if*, but *when*.

### Critical appraisal: limitations, failures, and lessons learned

While the preceding case studies demonstrate the transformative potential of artificial intelligence (AI) in natural product (NP) discovery, a balanced assessment must also acknowledge the substantial limitations, failures, and unresolved challenges that continue to constrain real-world impact. One persistent issue is the high false-positive rate associated with AI-guided screening campaigns. Despite impressive in silico performance, experimental validation consistently reveals that only a minority of computationally predicted “hits” exhibit confirmed bioactivity in wet-lab assays [157–159]. This disparity highlights fundamental limitations in current modeling approaches, particularly in capturing the complexity of biological systems and context-dependent molecular interactions. The discovery of halicin, although widely regarded as a milestone, further illustrates the gap between computational success and translational viability. Subsequent investigations have shown that halicin suffers from suboptimal pharmacokinetic properties, including poor absorption, rapid systemic clearance, and variable efficacy across infection models [160]. Notably, as of 2025, halicin has not advanced to clinical trials, underscoring that AI can accelerate early discovery stages but cannot bypass the inherent attrition of drug development. Another major limitation arises from domain shift, whereby AI models trained predominantly on synthetic, drug-like chemical libraries perform poorly when applied to structurally distinct NPs. The higher molecular weight, increased sp^3^ content, and pronounced stereochemical complexity of NPs often place them outside the applicability domain of conventional models, necessitating NP-specific datasets and architectures [161, 162]. Reproducibility also remains a concern, mirroring challenges seen across AI/ML research more broadly. Independent attempts to replicate AI-guided NP discoveries have yielded inconsistent outcomes, with variations in data preprocessing, hyperparameter selection, and random initialization significantly influencing model performance [163]. Finally, data quality remains a fundamental bottleneck, as NP datasets frequently suffer from incomplete stereochemical annotation, scarcity of reported negative results, and batch effects arising from heterogeneous assay conditions [164]. Collectively, these challenges do not diminish AI’s transformative promise but instead emphasize the need for realistic expectations, rigorous validation, and sustained interdisciplinary collaboration. The translation of AI-predicted NP hits into clinically approved therapeutics remains a complex and uncertain journey, demanding continued methodological refinement and close integration between computation and experiment.

## Future roadmap: AI–natural product coevolution in the era of emerging technologies

The future of NP research is poised to evolve alongside advances in AI and a new generation of enabling technologies. As conventional AI tools become embedded within the NP discovery pipeline, forward-looking researchers must embrace a broader, more integrated technological landscape—one that includes quantum machine learning (QML), federated data sharing, AI-guided synthetic biology, and progressive policy frameworks to ensure ethical and sustainable innovation (Table 7).

**Table 7:** Emerging technologies shaping the future of AI–natural product discovery

Technology	Description	Application in NP research	Current limitations	Timeline for maturity
Quantum–AI synergy	Integration of quantum computing with AI/ML to solve computationally intensive molecular problems with quantum-level precision	Accurate simulation of NP structure, reaction energetics, transition states, and quantum properties for complex bioactive leadst	Immature hardware, barren plateau problem, limited scalability, high cost, and unclear short-term clinical utilityt	Long-term (10–15 years)
Federated learning (FL)	Privacy-preserving, decentralized machine learning approach that enables collaborative model training without sharing raw data	Integrates NP data across academic, clinical, and industrial silos; enables inclusive models representing global chemical diversity	Communication overhead, lack of modularity, and difficulty maintaining model performance across heterogeneous datasets	Near- to mid-term (2–5 years)
Generative AI / LLMs	Large models trained on chemical and textual corpora to generate novel compounds, predict targets, or translate traditional medicine texts	De novo NP-inspired compound generation, target prediction, bioactivity annotation, and mining traditional medical knowledge	Data bias, hallucination, interpretability issues, limited NP-specific fine-tuning	Mid-term (3–7 years)
AI for molecular de-extinction	Application of generative models to predict and resurrect compounds from extinct proteomes or metabolomes	Expands the chemical space beyond extant species; enables the rediscovery of unique bioactive scaffolds lost to evolution	Requires reliable reconstruction of ancient sequences, validation bottlenecks, and ethical considerations	Early-stage, mid- to long-term
NLP for ethnopharmacology	Natural language processing applied to mine, interpret, and standardize knowledge from traditional medicine records	Unlocks underutilized historical NP data, supports modernization of traditional systems, and supports inclusive AI models	Terminology inconsistency, cultural context ambiguity, and lack of high-quality labeled corpora	Ongoing; mature in 3–5 years

In this roadmap, we begin by exploring the synergistic potential of quantum computing and AI—a convergence uniquely suited to address the structural, electronic, and energetic complexity of NP molecules. While still in its infancy, this emerging frontier promises to unlock new levels of molecular modeling fidelity and chemical insight that are currently beyond reach.

### Quantum–AI synergy for modeling molecular complexity

The intersection of quantum computing (QC) and artificial intelligence represents a disruptive opportunity to overcome long-standing limitations in NP-based drug discovery. Unlike classical algorithms, quantum algorithms are intrinsically suited to model the quantum mechanical nature of molecules—offering the potential to simulate electron interactions, reaction energetics, and conformational landscapes with unprecedented accuracy.

A landmark demonstration of quantum advantage came from Google’s 53-qubit quantum processor, which reportedly solved a problem in 200 s that would take a conventional supercomputer an estimated 10,000 years—underscoring the potential of quantum acceleration for scientific applications [165, 166]. In NP chemistry, such capabilities could enable quantum-level insight into complex scaffolds, stereoelectronic effects, and transition states that are notoriously difficult to model using classical techniques.

Several recent studies have begun to demonstrate the feasibility of this vision. Quantum circuit Born machines (QCBMs) have been employed to augment de novo molecular design, including the prediction of novel inhibitors for KRAS—a target historically considered “undruggable” [167]. In another example, Li et al. applied a variational quantum eigensolver (VQE) to estimate the Gibbs free energy barrier for the bioactive NP β-lapachone, achieving results comparable to density functional theory (DFT) simulations [168]. These early demonstrations suggest that hybrid quantum–AI models could eventually outperform classical methods in key tasks such as structure prediction, conformational sampling, and lead optimization.

However, significant technical and conceptual hurdles remain. One pressing challenge is the “dequantization trap”—a scenario in which quantum algorithms are restructured into classical analogs with comparable efficiency, thereby nullifying the quantum advantage [169]. Moreover, current quantum hardware is still highly susceptible to environmental noise, decoherence, and gate errors, making it unreliable for complex tasks [170, 171]. Estimates suggest that fault-tolerant, error-corrected quantum computers—with millions of stable qubits—are at least a decade away from practical implementation [172, 173].

From an algorithmic perspective, the barren plateau phenomenon poses a critical barrier for scalable quantum neural networks (QNNs). As QNNs grow in size, their optimization landscapes can become exponentially flat, resulting in vanishing gradients that make training infeasible—especially in high-dimensional chemical systems such as natural products [174]. Cost and integration are also practical concerns. Even classical DFT methods, despite their accuracy, are often too computationally expensive for use in high-throughput settings in pharmaceutical pipelines. This same cost barrier will likely apply to early implementations of quantum-enhanced methods until more efficient hybrid solutions are developed [175].

Despite these limitations, the long-term promise of quantum–AI integration remains profound. In the near future, a hybrid computational architecture may emerge, wherein classical AI performs large-scale screening, clustering, and predictive modeling, while quantum computers are reserved for specialized tasks—such as quantum property calculations, transition state optimization, or simulating reaction pathways involving heavy electron correlation.

Investing in this convergence today ensures that NP drug discovery pipelines will be ready to leverage quantum acceleration as soon as the technology reaches maturity. As QC, AI, and cheminformatics coevolve, they will form the foundation of a future in which nature-inspired drug discovery is not only faster and more accurate—but fundamentally redefined.

### Federated learning ecosystems: enabling secure collaboration in NP discovery

Federated learning (FL) offers a transformative solution to one of the most persistent bottlenecks in AI-driven NP research: fragmented, inaccessible, and siloed datasets. By allowing multiple institutions—such as pharmaceutical companies, academic laboratories, and clinical research centers—to collaboratively train machine learning models without sharing proprietary or sensitive data, FL preserves data privacy while enhancing the analytical reach and robustness of AI models [176]**.**

Originally introduced by McMahan et al. in 2017 for privacy-preserving mobile applications [177], FL has since gained traction in regulated domains like healthcare, where data centralization is infeasible due to privacy concerns and ethical constraints [178]. In NP drug discovery, similar challenges abound. Datasets are often heterogeneous, inconsistently annotated, and geographically or institutionally isolated. FL directly addresses this issue by enabling decentralized training on these disparate data sources, generating a globally shared model that reflects the full chemical, biological, and ethnopharmacological diversity of NP datasets—without requiring raw data exchange [179].

This distributed approach is particularly advantageous in NP contexts, where datasets often lack uniform metadata on chemical structure, bioactivity, pharmacokinetics, and toxicity. Through federated collaboration, organizations managing microbial libraries, traditional medicine repositories, or metabolomics datasets can contribute to model development while retaining full control over their underlying data. This yields more robust and generalizable AI models, capable of improving predictions related to bioactivity, drug-likeness, or therapeutic relevance—particularly in low-resource or underrepresented domains of natural product chemistry.

Real-world implementations of FL have already demonstrated superior predictive performance in pharmaceutical contexts. One example involves the use of a federated student–teacher architecture, where local models ("teachers") trained on private datasets transferred distilled knowledge to a global "student" model. The resulting federated student model outperformed all local models, displaying a wider applicability domain and greater predictive accuracy across heterogeneous datasets [179]. Such architectures can play a pivotal role in NP research by leveraging multi-omics data—including genomics, proteomics, metabolomics, and spatial transcriptomics—to uncover mechanisms of action, identify molecular targets, and guide compound prioritization for bioactive natural products.

Federated learning also holds the potential to democratize AI model development by ensuring that region-specific or endemic chemical space—such as plant-derived secondary metabolites from biodiversity hotspots or microbiome-sourced natural products—are adequately represented in global prediction pipelines. This is crucial, given the historical underrepresentation of traditional medicine systems and geographically unique NP libraries in many current datasets.

Nonetheless, FL presents several technical challenges that must be addressed to support widespread adoption. These include optimizing knowledge distillation methods to reduce communication overhead and ensure privacy-preserving updates, as well as developing modular model architectures capable of continuous learning without catastrophic forgetting [180]. Moreover, maintaining model performance across highly heterogeneous and unevenly distributed datasets remains an open research problem, especially in domains as complex and data-sparse as NP drug discovery.

In sum, federated learning provides a scalable, privacy-preserving, and ethically aligned framework for transforming fragmented NP data into shared computational assets. By enabling secure and decentralized model training across institutional boundaries, FL paves the way for more inclusive, accurate, and generalizable AI models. As NP research becomes increasingly globalized, federated ecosystems will likely be essential for building predictive pipelines that reflect the full breadth of natural product diversity and therapeutic potential.

### Translational accelerators: strategic partnerships and policy frameworks

Realizing the full potential of AI-integrated NP drug discovery will require more than technical innovation—it demands the formation of strategic partnerships, the development of pre-competitive consortia, and the implementation of adaptive policy frameworks. The complexity, cost, and interdisciplinarity inherent to AI-enhanced NP research—especially in areas such as computational NP design, federated data modeling, and biosynthetic pathway engineering—necessitate collaborative models that align resources, expertise, and infrastructure. These collective efforts are essential to de-risk high-impact innovation and to scale discovery from algorithmic predictions to therapeutic realities.

A number of public–private initiatives are already leading this transformation. The Accelerating Therapeutics for Opportunities in Medicine (ATOM) Consortium, the Machine Learning Ledger Orchestration for Drug Discovery (MELLODDY) project, and Target 2035 exemplify cross-sector collaborations that bring together pharmaceutical companies, academic institutions, and regulatory agencies to pool chemical, bioactivity, and clinical data for the development of next-generation AI models [181]. These consortia highlight the value of shared infrastructure and data harmonization in overcoming the fragmentation that has historically hindered natural product research and drug discovery more broadly.

On the regulatory front, agencies are increasingly proactive in facilitating the responsible integration of AI and ML tools into biomedical development pipelines. The U.S. Food and Drug Administration (FDA), for instance, has organized a series of workshops and issued evolving guidance documents—most notably its “AI/ML for Drug Development Discussion Paper,” originally published in May 2023 and revised in February 2025. This document outlines key principles related to model transparency, validation, adaptability, and post-deployment performance monitoring. Concurrently, the European Medicines Agency (EMA), in collaboration with the Heads of Medicines Agencies (HMA), has launched a multi-year artificial intelligence workplan (2023–2028) aimed at standardizing the use of AI in regulatory science. This initiative focuses on improving clinical trial design, enhancing pharmacovigilance systems, and embedding AI into regulatory decision-making processes.

Together, these public–private alliances and policy initiatives are laying the groundwork for a more interoperable, ethically governed, and innovation-ready NP discovery ecosystem. By standardizing data formats, enforcing model explainability, and enabling equitable access to both resources and results, such frameworks will accelerate the path from AI-predicted NP hits to clinically validated leads.

### Global health equity: targeting neglected diseases through AI–NP integration

Beyond the frontiers of technological advancement and commercial innovation, the convergence of AI and NP research must also be steered toward global health equity—particularly in addressing neglected tropical diseases (NTDs). Affecting more than one billion people, primarily in low- and middle-income countries, NTDs remain critically underfunded and underserved in the pharmaceutical pipeline, despite their profound public health burden. Historically, natural products have yielded a wealth of antiparasitic and antimicrobial agents, many of which remain underexplored. This latent therapeutic potential offers a strategic opportunity to re-engage NP discovery in the fight against NTDs.

AI has the capacity to democratize access to NP-based discovery by lowering both economic and technical barriers that typically hinder research in resource-limited settings. ML models can rapidly screen, annotate, and prioritize compounds from expansive NP libraries, enabling efficient hit-to-lead workflows at a fraction of the traditional cost. The Drugs for Neglected Diseases initiative (DNDi) has emphasized the transformative value of ML in this context—not only for its scalability and speed, but also for its accessibility, empowering researchers in the Global South to participate in advanced computational discovery efforts [182].

One illustrative example comes from the work of Gaudry and colleagues, in collaboration with the Swiss Tropical and Public Health Institute (Swiss TPH) and DNDi, who deployed a semantic AI pipeline to analyze a library of 1,600 plant extracts screened against *Trypanosoma* species. The system successfully annotated both known and previously uncharacterized antiprotozoal compounds, accelerating the early discovery pipeline and demonstrating how AI can unlock hidden potential within underutilized ethnopharmacological collections [183]. Comparable frameworks have also been used to repurpose archived NP-derived drugs for novel NTD indications, reinforcing the broader utility of AI in extending the therapeutic relevance of existing natural compound libraries.

To ensure sustainable impact, future AI–NP initiatives targeting NTDs must be grounded in principles of open science, equitable data sharing, and socially responsible licensing. Current NTD-focused consortia are increasingly embracing transparent methodologies, public availability of training datasets, and benefit-sharing models that acknowledge and protect the contributions of local communities—especially when indigenous knowledge and biodiversity are central to discovery efforts. The integration of federated learning, generative AI, and quantum simulation under these ethical frameworks may further accelerate innovation while upholding fairness and inclusivity.

Ultimately, the convergence of AI and NP research presents not just an opportunity for scientific advancement but a mandate for global responsibility. By directing these powerful tools toward neglected diseases, the scientific community can help close critical treatment gaps and deliver innovative, locally relevant, and globally impactful solutions for some of the world’s most underserved populations.

### A proposed framework for AI-NP coevolutionary discovery

**Fig. 4:**
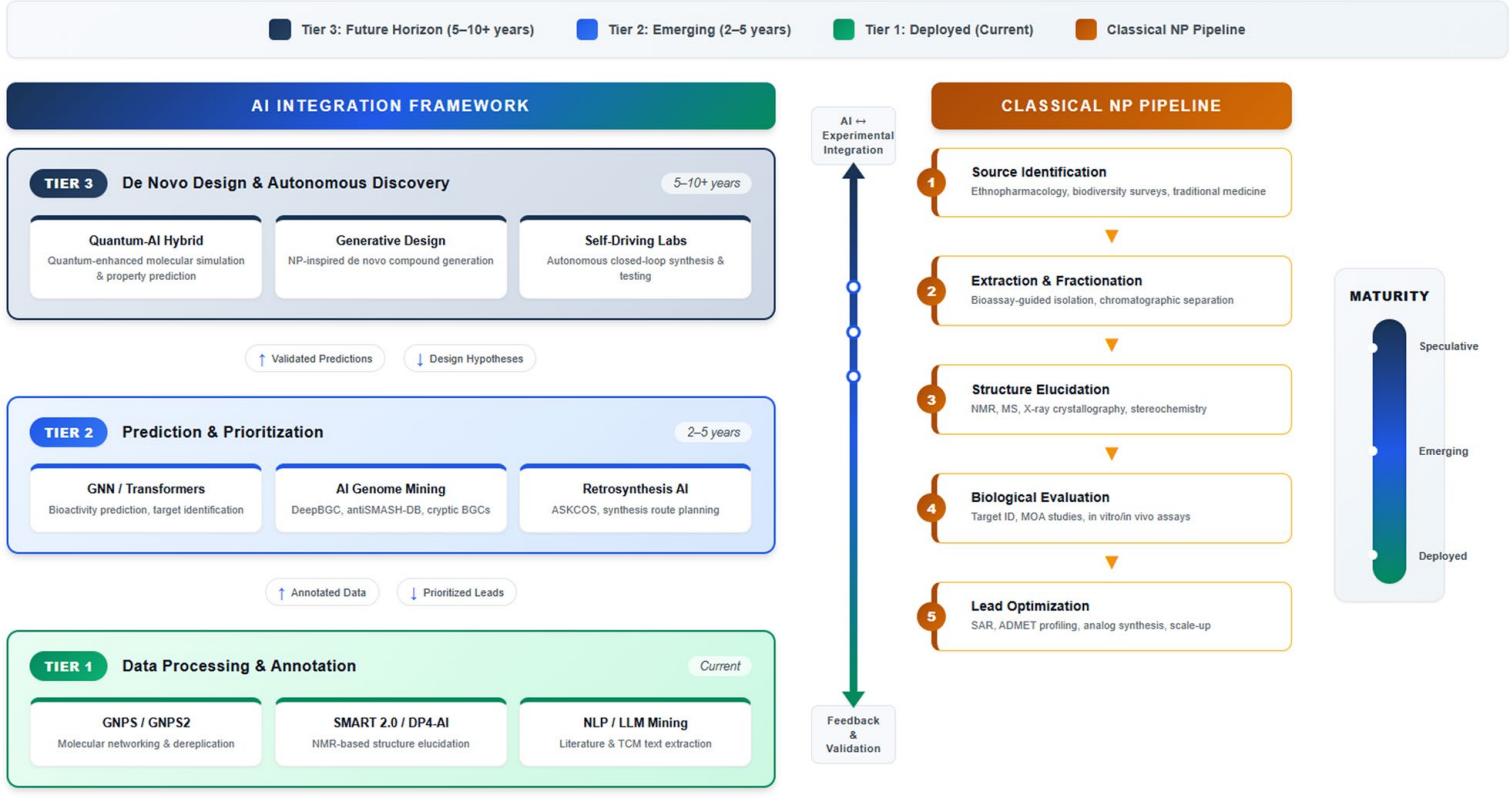
Proposed three-tier framework for AI integration in natural product drug discovery. The framework maps AI applications across the discovery pipeline, from mature data processing tools (Tier 1), through emerging predictive applications (Tier 2), to future autonomous discovery systems (Tier 3). Arrows indicate data flow and feedback loops between computational and experimental components

Drawing from the technologies, case studies, and challenges discussed throughout this review, we propose a three-tier integration framework for AI in NP discovery (Fig. 4):

#### Tier 1—AI for data processing and annotation (current maturity)

At this foundational level, AI tools address data management challenges including automated spectral annotation, dereplication, and literature mining. Technologies like GNPS molecular networking, SMART 2.0 NMR analysis, and NLP-based extraction from traditional medicine texts represent mature applications ready for routine deployment.

#### Tier 2—AI for prediction and prioritization (emerging applications)

Building on processed data, Tier 2 applications include bioactivity prediction, target identification, and lead prioritization. GNN-based property prediction, multi-task models for polypharmacology assessment, and AI-guided genome mining fall within this category. While promising, these tools require continued validation and domain adaptation for NP-specific applications.

#### Tier 3—AI for de novo design and autonomous discovery (future horizon)

The most ambitious tier envisions AI systems capable of designing novel NP-inspired compounds, predicting optimal biosynthetic routes, and potentially guiding autonomous robotic synthesis and testing platforms. Quantum-AI hybrid approaches and fully integrated self-driving laboratories represent this frontier, likely requiring 5–10 years for practical implementation.

### Key enabling factors

Realizing this framework requires: (1) standardized, FAIR-compliant NP databases with complete stereochemical and bioactivity annotation; (2) federated learning infrastructure enabling collaboration without compromising proprietary data; (3) validation benchmarks specifically designed for NP discovery tasks; and (4) interdisciplinary training programs producing researchers fluent in both NP chemistry and computational methods.

### Human-AI collaboration

Critically, we envision AI as augmenting rather than replacing human expertise. The irreducible complexity of NP discovery—encompassing ecological relationships, evolutionary optimization, cultural knowledge, and serendipitous observation—demands continued human insight. The most productive future lies in intelligent partnership, where AI handles data-intensive pattern recognition while human researchers contribute creativity, contextual judgment, and ethical oversight.

## Conclusion

From ancient medical traditions to modern pharmaceutical research, natural products (NPs) have consistently shaped the foundations of therapeutic innovation. Their structural complexity, evolutionary optimization, and intrinsic biological relevance have made them indispensable as both direct medicines and as scaffolds for modern drug design. Nevertheless, despite this enduring value, NP-based drug discovery has gradually lost prominence over recent decades, owing to methodological bottlenecks, scalability constraints, frequent rediscovery, and the growing dominance of synthetic and computationally driven discovery paradigms. Paradoxically, many contemporary “synthetic” drugs continue to draw heavily from NP-inspired chemical space, underscoring the persistent relevance of nature-derived molecular architectures.

Crucially, the therapeutic potential of natural products remains far from exhausted. Earth’s biosphere continues to represent an immense and largely untapped reservoir of chemical diversity, spanning terrestrial, marine, and microbial ecosystems. However, the scale, contextual richness, and structural intricacy of this diversity increasingly exceed the analytical capacity of classical discovery pipelines. Artificial intelligence offers a powerful methodological response to these challenges. By enabling high-dimensional pattern recognition, integrative learning across heterogeneous data types, and scalable prediction, AI facilitates more systematic exploration of NP chemical space—accelerating dereplication, metabolite annotation, target identification, and lead prioritization while reducing inefficiencies inherent to traditional workflows.

Although artificial intelligence has existed conceptually for decades, only recent advances in computing infrastructure, algorithmic design, and data availability have enabled its practical deployment across the NP discovery pipeline. As reviewed here, AI methods are now contributing meaningfully at multiple levels, from text mining of ethnopharmacological knowledge and genome mining of cryptic biosynthetic pathways to AI-assisted structural elucidation, retrosynthetic planning, and predictive pharmacology. Emerging approaches—including federated learning, multimodal AI, and quantum-informed modeling—offer additional opportunities to extend these capabilities, particularly by enabling collaborative discovery while preserving data sovereignty and addressing the molecular complexity characteristic of NPs.

At the same time, this review emphasizes that AI is not a panacea. High false-positive rates, domain-shift limitations, data quality issues, and reproducibility concerns remain significant barriers to translation. Progress will therefore depend not on algorithmic sophistication alone, but on rigorous validation, NP-specific benchmarks, standardized data practices, and sustained experimental integration. The future of NP-based therapeutics will be shaped by interdisciplinary collaboration—uniting pharmacognosy, chemistry, biology, data science, and ethics—to ensure that AI applications are both scientifically robust and socially responsible.

In synthesizing historical perspective, current advances, and future directions, this review presents AI not merely as a supporting tool, but as a complementary discipline capable of addressing long-standing bottlenecks in NP research when applied judiciously. The next phase of drug discovery is unlikely to arise from synthetic ingenuity or computational power in isolation. Rather, it will emerge from a progressive integration of natural product science and artificial intelligence—an informed coevolution that leverages the strengths of both to expand the global pharmacopeia and improve therapeutic innovation in an equitable and sustainable manner.

## Supplementary Information

Supplementary file 1.

## Data Availability

Data sharing is not applicable to this article, as no new data were generated or analysed in this study.
