# Supplementary material for: Nature meets machine: the AI renaissance in natural product drug discovery
Source: Nat Prod Bioprospect. 2026 Mar 2;16(1):37. doi: 10.1007/s13659-025-00589-6 (PMC12953908; doi:10.1007/s13659-025-00589-6)
Supplement: Supplementary file 1 [file 13659_2025_589_MOESM1_ESM.docx]

***Supplementary Table S1****. Chemical structures of key natural products and AI-discovered compounds discussed in this review.*

| **S.No** | **Name** | **Structure/Peptide Sequence** | **Description** |
| --- | --- | --- | --- |
| 1 | Halicin |  | A structurally novel, broad-spectrum antibiotic identified using deep learning–based screening. Halicin exhibits activity against multiple drug-resistant bacterial pathogens and represents a landmark example of AI-enabled antibiotic discovery. |
| 2 | Abaucin |  | A narrow-spectrum antibiotic discovered through AI-guided screening, specifically targeting *Acinetobacter baumannii*. Abaucin demonstrates the potential of AI to enable precision antimicrobial discovery with reduced off-target effects. |
| 3 | Paclitaxel |  | A plant-derived anticancer agent originally isolated from *Taxus brevifolia*. Paclitaxel stabilizes microtubules and remains a cornerstone of cancer chemotherapy; recent studies highlight AI-assisted optimization of its biosynthesis and dosing strategies. |
| 4 | Marinopyrrole A |  | A marine-derived natural product isolated from *Streptomyces* species. Marinopyrrole A has served as a scaffold for AI-guided derivatization, leading to the development of more potent and synthetically tractable anticancer analogues. |
| 5 | Artemisinin |  | A plant-derived antimalarial compound isolated from *Artemisia annua*. Artemisinin exemplifies the value of traditional knowledge and natural products in modern therapeutics and serves as a reference point for AI-enabled NP rediscovery and optimization. |
| 6 | Mammuthusin-2 | LILSLLINSTLALL | An antimicrobial peptide identified through AI-driven “molecular de-extinction” by mining extinct proteomes. Derived from the woolly mammoth (*Mammuthus primigenius*), Mammuthusin-2 exhibits potent antimicrobial activity in preclinical models. |
| 7 | Elephasin-2 | VFLTLNSIKVLKL | An AI-predicted antimicrobial peptide derived from the extinct straight-tusked elephant (*Elephas antiquus*). Elephasin-2 demonstrates strong antibacterial efficacy and highlights AI’s ability to access lost biochemical diversity. |
